# Human NK cell receptor KIR2DS4 detects a conserved bacterial epitope presented by HLA-C

**DOI:** 10.1073/pnas.1903781116

**Published:** 2019-05-28

**Authors:** Malcolm J. W. Sim, Sumati Rajagopalan, Daniel M. Altmann, Rosemary J. Boyton, Peter D. Sun, Eric O. Long

**Affiliations:** ^a^Molecular and Cellular Immunology Section, National Institute of Allergy and Infectious Diseases, National Institutes of Health, Rockville, MD 20852;; ^b^Structural Immunology Section, Laboratory of Immunogenetics, National Institute of Allergy and Infectious Diseases, National Institutes of Health, Rockville, MD 20852;; ^c^Lung Immunology Group, Department of Medicine, Imperial College London, London W12 0NN, United Kingdom

**Keywords:** NK cells, bacteria, KIR, HLA-C, RecA

## Abstract

Natural killer (NK) cells have an important role in immune defense against viruses and cancer. Activation of human NK cell cytotoxicity toward infected or tumor cells is regulated by killer cell immunoglobulin-like receptors (KIRs) that bind to human leukocyte antigen class I (HLA-I). Combinations of KIR with HLA-I are genetically associated with susceptibility to disease. KIR2DS4, an activating member of the KIR family with poorly defined ligands, is a receptor of unknown function. Here, we show that KIR2DS4 has a strong preference for rare peptides carrying a Trp at position 8 (p8) of 9-mer peptides bound to HLA-C*05:01. The complex of a peptide bound to HLA-C*05:01 with a Trp at p8 was sufficient for activation of primary KIR2DS4^+^ NK cells, independent of activation by other receptors and of prior NK cell licensing. HLA-C*05:01^+^ cells that expressed the peptide epitope triggered KIR2DS4^+^ NK cell degranulation. We show an inverse correlation of the worldwide allele frequency of functional *KIR2DS4* with that of HLA-C*05:01, indicative of functional interaction and balancing selection. We found a highly conserved peptide sequence motif for HLA-C*05:01–restricted activation of human KIR2DS4^+^ NK cells in bacterial recombinase A (RecA). KIR2DS4^+^ NK cells were stimulated by RecA epitopes from multiple human pathogens, including *Helicobacter*, *Chlamydia*, *Brucella*, and *Campylobacter.* We predict that over 1,000 bacterial species could activate NK cells through KIR2DS4, and propose that human NK cells also contribute to immune defense against bacteria through recognition of a conserved RecA epitope presented by HLA-C*05:01.

## Significance

Natural killer (NK) cells are known for their role in defense against viruses and cancer. Their activity is regulated, in part, by killer cell immunoglobulin-like receptors (KIRs) that bind to polymorphic human leukocyte antigen (HLA) class I molecules. The KIR family includes an activation receptor of unknown function, KIR2DS4. Here, we show that KIR2DS4 binding to HLA-C*05:01 is dependent on specific peptides that include a Trp at position 8 of 9-mer peptides associated with HLA-C*05:01. Through sequence homology, we identified a highly conserved peptide sequence in bacterial recombinase A that binds to HLA-C*05:01 and stimulates KIR2DS4^+^ NK cells. We predict that over 1,000 bacterial species contain this epitope and propose that NK cells contribute also to immune defense against bacteria.

Major histocompatibility complex class I (MHC-I) molecules play critical roles in innate and adaptive immunity. MHC-I molecules present short peptides, commonly 8–11 amino acids in length, which are surveilled by αβ T cell receptors expressed by CD8^+^ T cells. MHC-I also serves as a critical regulator of natural killer (NK) cells, innate immune cytotoxic cells with the capacity to produce proinflammatory cytokines (1, 2). Following the “missing self” hypothesis, MHC-I binding inhibitory receptors expressed by NK cells detect loss of MHC-I, leading to NK cell activation (3). Additionally, interactions between inhibitory receptors and MHC-I dictate the effector potential of NK cells via a process known as “education” or “licensing” (2, 4). NK cells have established roles in immune defense against cancers and viral infections, where loss or down-regulation of MHC-I is common (5, 6). The functions of MHC-I binding NK cell inhibitory receptors appear conserved across species and different families of receptors.

In humans, the major NK receptors for human leukocyte antigen class I (HLA-I) (human MHC-I) are CD94:NKG2A, which binds HLA-E, and the killer cell immunoglobulin (Ig)-like receptors (KIRs). There are 14 KIR genes which encode activating and inhibitory receptors. The ligands for inhibitory KIRs are well defined as groups of HLA-A, HLA-B, or HLA-C allotypes, each with a common epitope. All HLA-C allotypes carry either the C1 or C2 epitope, which are ligands for the inhibitory receptors KIR2DL2/3 and KIR2DL1, respectively (7). KIRs bind the peptide-exposed face of HLA-I toward the C-terminal end of the peptide, incorporating peptide into the binding site, and all HLA-C binding KIRs studied to date demonstrate a degree of peptide selectivity (8–13). In contrast to the inhibitory KIRs, definitive functional ligands for activating KIRs are still lacking.

The KIR genes are organized into two broad haplotypes, KIR A and KIR B, which differ by gene content. The simpler KIR A haplotype contains only one activating receptor *KIR2DS4*, while the B haplotype is characterized by variable gene content, including multiple activating receptor genes (7). The two haplotypes have a similar worldwide frequency but differ between populations, such that KIR A homozygotes are not rare and for whom *KIR2DS4* is the only activating KIR they carry. Due to variability of KIR haplotypes and the fact that HLA-I and KIR are on different chromosomes, individuals can express orphan receptors or ligands without the corresponding KIR. Consequently, gene association studies have linked the presence or absence or KIR and ligand pairs with many disease processes, including viral infections, autoimmunity, and cancer (7, 14–18). Additionally, activating KIRs with the ability to bind HLA-C appear to have a protective role against disorders of pregnancy (15, 19, 20).

The *KIR2DS4* locus is not fixed, and two major alleles exist that encode either the full-length receptor (KIR2DS4-fl) or a version with a deletion (KIR2DS4-del). KIR2DS4-del encodes a 22-base pair deletion, leading to an early stop codon creating a truncated soluble protein with no recorded HLA-I binding (21, 22). KIR2DS4-fl is an HLA-I binding receptor and binds a subset of C1 and C2 HLA-C allotypes in contrast to other KIR2D receptors, which dominantly bind C1 or C2 (22). This previous report identified KIR2DS4 ligands via a binding assay using soluble KIR molecules, and many HLA-A, HLA-B, and HLA-C proteins bound to beads (23). This method has proved useful to screen many HLA-I allotypes at once, but the sequence and diversity of peptides presented on the beads are unknown. Furthermore, it is not clear whether HLA-C constitutes a functional ligand for KIR2DS4 or how a peptide sequence contributes to KIR2DS4 binding. Indeed, the only known functional ligand for KIR2DS4 is HLA-A*11:02 (22).

Carrying KIR2DS4-fl is associated with protection from preeclampsia and glioblastoma, and with higher viral loads and faster progression to AIDS in HIV infection (19, 24–26). There is a clear need to define functional ligands for KIR2DS4 to fully understand its role in these disease processes and in the regulation of NK cells more generally. HLA-C*05:01, a C2 allotype, was reported to bind KIR2DS4-fl (22). Henceforth, we refer to KIR2DS4-fl as KIR2DS4 unless in direct comparison to KIR2DS4-del. The aim of this study was to establish whether HLA-C*05:01 is a functional ligand for KIR2DS4 and if peptide sequence influences KIR2DS4 binding. We find that KIR2DS4 binds HLA-C*05:01 in a highly peptide-selective manner and that this binding potently activates KIR2DS4^+^ NK cells. Further, we link peptide-specific recognition of HLA-C*05:01 by KIR2DS4 to a protein highly conserved among bacteria pathogenic in humans.

## Results

### Highly Selective Binding of KIR2DS4 to HLA-C*05:01 Loaded with a 9-Mer Peptide with Trp at Position 8.

**Fig. 1.**
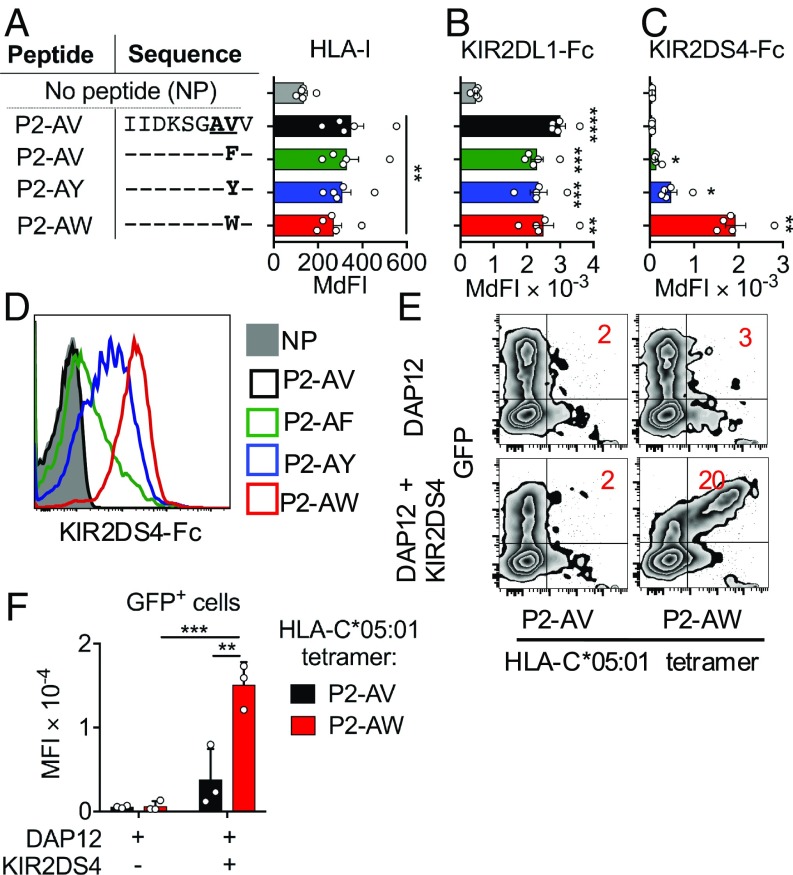
Binding of KIR2DS4 to HLA-C*05:01 loaded with a peptide with Trp at p8. (*A*) Stabilization of HLA-I expression on 221-C*05:01-ICP47 cells incubated overnight at 26 °C with 100 μM peptides P2-AV, P2-AF, P2-AY, and P2-AW. Amino acids identical to the P2-AV sequence are indicated with –. Data are shown as median fluorescence intensity (MdFI) (*n* = 5). KIR2DL1-Fc (*B*) and KIR2DS4-Fc (*C*) binding to 221-C*05:01-ICP47 cells incubated with peptides as shown in *A* or no peptide (NP) is illustrated (*n* = 5). (*D*) Flow cytometry histograms displaying KIR2DS4-Fc binding to 221-C*05:01-ICP47 cells from a representative experiment, from a total of five independent experiments. (*E*) Flow cytometry biplots displaying HLA-C*05:01–tetramer binding to 293T cells transfected with separate vectors (pIRES2-eGFP) containing cDNA encoding DAP12 and KIR2DS4 or DAP12 only. HLA-C*05:01 tetramers were refolded with P2-AV or P2-AW. Data are from one representative experiment out of three independent experiments. (*F*) HLA-C*05:01–tetramer binding to 293T cells as in *E*, displayed as MFI, from three independent experiments. Statistical significance was assessed by one-way ANOVA (*A*–*C*) with Tukey’s multiple comparisons test and two-way ANOVA (*F*) with Dunnett’s multiple comparisons test (*P < 0.05, **P < 0.01, ***P < 0.001, ****P < 0.0001). In *A*–*C*, significance is indicated in comparison to NP.

Binding of a soluble KIR2DS4–IgG1-Fc fusion protein (KIR2DS4-Fc) was tested with the HLA-I–deficient cell line 721.221 (221) transfected with HLA-C*05:01 (221-C*05:01) or HLA-C*04:01 (221-C*04:01) (*SI Appendix*, Fig. S1*A*). While KIR2DL1-Fc bound both cell lines strongly, no binding to KIR2DS4-Fc was detected. KIR2DL1 bound HLA-C*05:01 in the context of many different peptide sequences (10). To test whether KIR2DS4 binding may be more peptide-specific, we examined KIR2DS4-Fc binding to HLA-C*05:01 loaded with individual peptide sequences, using a transporter associated with antigen presentation (TAP)-deficient cell line expressing HLA-C*05:01, as described (10). We tested 46 “self” peptides previously eluted from purified HLA-C*05:01; none conferred binding of KIR2DS4-Fc to HLA-C*05:01 (*SI Appendix*, Fig. S1 *B* and *C*). However, “self” peptide P2 (IIDKSGSTV) substituted with Ala and Tyr at position 7 (p7) and p8 (P2-AY) conferred strong KIR2DS4-Fc binding to HLA-C*05:01 (*SI Appendix*, Fig. S1 *B* and *C*). P2-AY was synthesized as part of a screen to test the impact of the peptide sequence at p7 and p8 on inhibitory KIR binding (10). Nine additional amino acid substitutions at p7 and p8 of peptide P2 failed to bind to KIR2DS4, which suggested a contribution of the p8 Tyr. HLA-C*08:02 is a C1 allotype that differs from HLA-C*05:01 by only the C1 and C2 epitopes (amino acids at p77 and p80), and it is stabilized by peptides eluted from HLA-C*05:01 (10). However, KIR2DS4 did not bind HLA-C*08:02 when loaded with P2-AY, suggesting the C2 epitope positively contributed to KIR2DS4 binding to HLA-C*05:01 (*SI Appendix*, Fig. S1*D*). As Tyr is a large aromatic residue, we tested two other large aromatic residues, Phe (P2-AF) and Trp (P2-AW), at p8 and used Val as a control (P2-AV; Fig. 1*A*). All four peptides stabilized HLA-C*05:01 on 221-C*05:01-ICP47 cells to a similar extent and conferred strong KIR2DL1-Fc binding (Fig. 1 *A* and *B*). Substitution of p8 Tyr to Phe reduced KIR2DS4-Fc binding, while substitution to Trp substantially increased binding of KIR2DS4-Fc (Fig. 1 *C* and *D*). To confirm this interaction, soluble HLA-C*05:01 that was refolded with peptide P2-AV or P2-AW was produced as tetramers. Tetramers bound similarly to 293T cells transiently transfected with KIR2DL1 (*SI Appendix*, Fig. S1*F*). In contrast, only HLA-C*05:01 tetramers refolded with P2-AW–bound cells transfected with KIR2DS4 and DAP12 (Fig. 1 *E* and *F*). Negligible binding of HLA-C*05:01 tetramers occurred with cells transfected with DAP12 alone. Collectively, these data show that KIR2DS4 binds HLA-C*05:01 with a high peptide selectivity. Of 61 different peptide sequences tested, only P2-AF, P2-AY, and P2-AW conferred measurable KIR2DS4 binding to HLA-C*05:01.

### Peptide-Specific Activation of KIR2DS4^+^ NK Cells by HLA-C*05:01.

We next tested the capacity of peptide/HLA-C*05:01 complexes to activate KIR2DS4^+^ NK cells in degranulation assays. Primary resting NK cells from KIR2DS4^+^ donors were mixed with 221-C*05:01-ICP47 cells loaded with peptide P2-AV, P2-AF, P2-AY, or P2-AW. To eliminate the contribution of KIR2DL1/S1 binding to HLA-C*05:01, NK cells were gated as CD56^dim^ and KIR2DL1/S1^−^ (EB6). KIR2DS4^+^, but not KIR2DS4^−^, NK cells displayed increased degranulation in response to cells loaded with P2-AY and P2-AW (*SI Appendix*, Fig. S2 *A* and *B*).

**Fig. 2.**
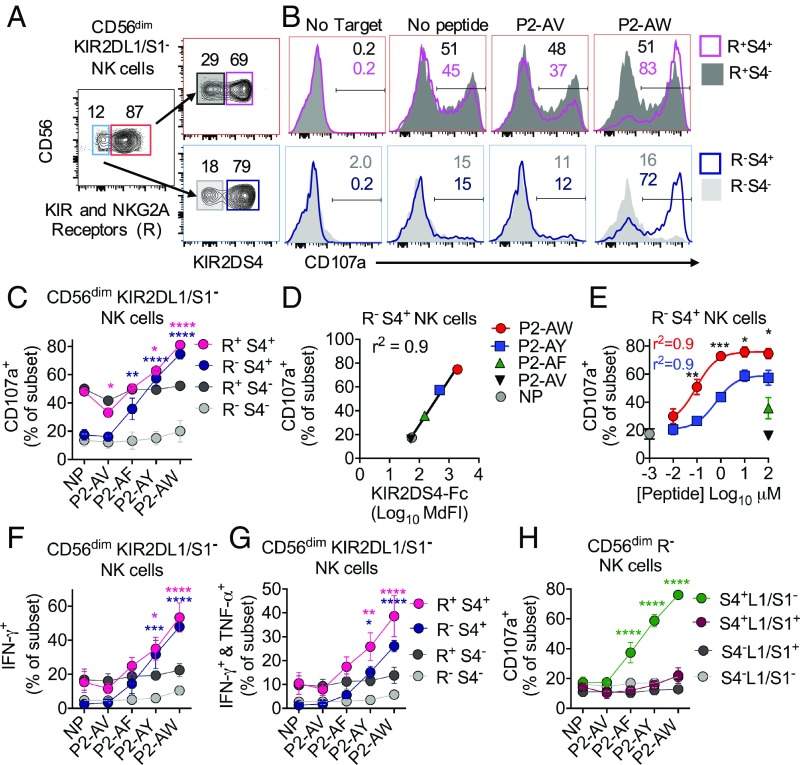
Activation of KIR2DS4^+^ NK cells by peptide/HLA-C*05:01. (*A*) Gating strategy to identify four NK cell subsets by expression of KIR2DS4 (S4) and MHC-I binding receptors (R) within the CD56^dim^ KIR2DL1/S1^−^ NK subset. The R^+^ subset includes NK cells positive for KIR2DL2/L3/S2 (clone GL183), KIR3DL1/3DS1 (clone Z27), and NKG2A (clone Z199). (*B*) Flow cytometry histograms displaying CD107a expression on NK cell subsets defined in *A* in response to 221-C*05:01-ICP47 cells loaded with P2-AV, P2-AW, no peptide, or no target cells. Data are from one representative experiment out of three independent experiments. (*C*) Expression of CD107a on NK cell subsets as in *B* in response to 221-C*05:01-ICP47 cells loaded with P2-AV, P2-AF, P2-AY, P2-AW, or no peptide (NP). Data are mean and SEM from three independent experiments with NK cells from different donors. (*D*) Expression of CD107a on R^−^S4^+^ NK cells from *C* is correlated with KIR2DS4-Fc binding to 221-C*05:01-ICP47 cells from Fig. 1*D*, shown as log_10_ median fluorescence intensity (MdFI) (Pearson correlation: *r*^2^ = 0.9, *P* = 0.0003). (*E*) Expression of CD107a on R^−^S4^+^ NK cells in response to 221-C*05:01-ICP47 cells loaded with increasing concentrations of P2-AY and P2-AW. R^−^S4^+^ NK cell responses to 221-C*05:01-ICP47 cells loaded with 100 μM peptides P2-AF and P2-AV or without peptide are also shown. Samples are color-coded as in *D*. Data are mean and SEM from three independent experiments with NK cells from different donors. Expression of IFN-γ only (*F*) and coexpression of IFN-γ and TNF-α (*G*) are shown on NK cell subsets as defined in *A* in response to 221-C*05:01-ICP47 cells loaded with P2-AV, P2-AF, P2-AY, P2-AW, or NP. Data are mean and SEM from three independent experiments with NK cells from different donors. (*H*) Expression of CD107a on R^−^ NK cells in response to 221-C*05:01-ICP47 cells loaded with P2-AV, P2-AF, P2-AY, P2-AW, or NP. R^−^ NK cells were gated into four populations based on the expression of KIR2DS4 (S4) and KIR2DL1/S1 (L1/S1). Data are mean and SEM from three independent experiments with NK cells from different donors. Statistical significance was assessed by two-way ANOVA with Dunnett’s multiple comparisons test (*P < 0.05, **P < 0.01, ***P < 0.001, ****P < 0.0001). In *C*, *G*, and *H*, significance is indicated in comparison to NP and is color-coded by NK cell subsets. In *E*, significance is indicated in comparing P2-AW and P2-AY.

Due to the variegated expression of inhibitory NKG2A and KIRs, KIR2DS4^+^ and KIR2DS4^−^ NK cells include both licensed and unlicensed NK cells. Licensing endows NK cell subsets with greater capacity to degranulate in the absence of MHC-I. However, activation of KIR2DS4^+^ NK cells requires expression of MHC-I; thus, it is not clear how NK cell licensing may impact activation of KIR2DS4^+^ NK cells. To examine the role of NK cell licensing on activation of KIR2DS4^+^ NK cells, we gated CD56^dim^ KIR2DL1/S1^−^ NK cells into four subsets based on the expression of KIR2DS4 (S4) and other MHC-I binding receptors (KIR2DL2/L3/S2, NKG2A, and KIR3DL1/S1), termed receptor-positive (R^+^) and receptor-negative (R^−^) (Fig. 2*A*). All donors carry the ligand for NKG2A (HLA-E), while donors vary for the presence of the ligands for KIR3DL1 and KIR2DL2/3. Thus, R^+^ NK cells are expected to be licensed and degranulate well in response to 221-C*05:01-ICP47 cells as they lack ligands for KIR2DL2/3, NKG2A, and KIR3DL1. R^−^ NK cells lack all licensing receptors and are expected to degranulate weakly in response to 221-C*05:01-ICP47 cells. As expected, licensed R^+^ NK cells degranulated well in response to 221-C*05:01-ICP47 cells, while the R^−^ NK cells degranulated poorly irrespective of KIR2DS4 expression (Fig. 2 *B* and *C*). In contrast, KIR2DS4^+^ NK cells degranulated strongly in response to cells loaded with P2-AW (Fig. 2*B*). As expected, cells loaded with P2-AV did not stimulate KIR2DS4^+^ NK cells, while P2-AF and P2-AY did stimulate KIR2DS4^+^ NK cells to a lesser extent than P2-AW (Fig. 2 *B* and *C*). Remarkably, the potent activation of KIR2DS4^+^ NK cells in response to cells loaded with P2-AW was similar between R^+^ and R^−^ subsets. Thus, even unlicensed R^−^ NK cells can respond strongly to KIR2DS4 stimulation, overriding the lack of licensing.

There was a strong correlation (*r*^2^ = 0.9) between peptide-dependent KIR2DS4-Fc binding to HLA-C*05:01 and the functional response of R^−^S4^+^ NK cells in response to the same peptides (Fig. 2 *C* and *D*). Activation of R^−^S4^+^ NK cells in response to 221-C*05:01-ICP47 cells loaded with P2-AW and P2-AY was dependent on peptide concentration (Fig. 2*E*). Concentrations of peptide P2-AW lower than 100 nM activated R^−^S4^+^ NK cells, and half-maximal activation was reached with 0.07 μM P2-AW and 1 μM P2-AY (Fig. 2*E*). R^−^S4^+^ NK cells in response to 221-C*05:01-ICP47 cells loaded with P2-AW also reached a greater maximum than that obtained with high concentrations of P2-AY (Fig. 2*E*). Peptide-specific activation of KIR2DS4^+^ NK cells also induced production of IFN-γ and TNF-α, as ascertained by intracellular cytokine staining (Fig. 2 *F* and *G* and *SI Appendix*, Fig. S2*C*).

Signaling by immunoreceptor tyrosine-based inhibition motif-bearing inhibitory receptors dominates over signaling by NK cell activation receptors (2). The potent activation of NK cells by KIR2DS4, which can override the lack of NK cell licensing, suggested it may be difficult to inhibit. To test whether NK cell activation by KIR2DS4 is regulated by inhibitory receptors, we first regated our data from previous degranulation experiments (Fig. 2*C*). We gated NK cells as CD56^dim^ and then gated on R^−^ NK cells without excluding the KIR2DL1/S1^+^ (EB6) population. These NK cells were then gated into four subsets based on expression of KIR2DS4 (S4) and KIR2DL1/S1 (L1/S1) (Fig. 2*H*). S4^+^L1/S1^−^ NK cells displayed strong activation in response to 221-C*05:01-ICP47 cells loaded with P2-AF, P2-AY, and P2-AW (Fig. 2*H*). In contrast, S4^+^L1/S1^+^ NK cells displayed no activation even in response to cells loaded with P2-AW. Because the monoclonal antibody (mAb) EB6 binds KIR2DL1 and KIR2DS1, we confirmed these results with two donors who were typed to only carry KIR2DL1 (*SI Appendix*, Fig. S2 *D* and *E*). As KIR2DS4 is generally expressed by a higher proportion of NK cells than KIR2DL1, many KIR2DS4^+^ NK cells will not be inhibited (*SI Appendix*, Fig. S2 *F* and *G*). Together, these data show that KIR2DS4^+^ NK cells are potently activated to degranulate and produce cytokines upon engagement of KIR2DS4 and HLA-C*05:01 in a highly peptide-selective manner. Activation of KIR2DS4^+^ NK cells was limited to the KIR2DL1^−^ subset due to dominant inhibition by KIR2DL1^+^ NK cells. R^−^S4^+^ NK cells were unlicensed, but potently activated by KIR2DS4 stimulation overriding a lack of NK cell licensing. Therefore, under physiological conditions where the ligands for many NK cell inhibitory receptors are expressed, it is likely that the R^−^S4^+^ NK subset is the major operative subset due to its lack of inhibitory receptor expression.

### Peptide/HLA-C Is Sufficient to Activate KIR2DS4^+^ NK Cells.

Cross-linking of NK cell activation receptors is not sufficient to trigger resting NK cells, as activation receptors function as synergistic pairs (27–29). FcγRIIIA (CD16) is the only exception. To test the requirements for activation of KIR2DS4^+^ NK cells, we performed redirected antibody-mediated degranulation assays with FcR^+^ P815 cells. Minimal NK cell responses were observed when resting NK cells were preincubated with mAbs to either NKp46 or 2B4 and then mixed with P815 cells (*SI Appendix*, Fig. S3 *A* and *B*). In contrast, strong activation was observed when resting NK cells were preincubated with mAbs to both 2B4 and NKp46 (*SI Appendix*, Fig. S3 *A* and *B*). Remarkably, stimulation of KIR2DS4 alone was sufficient to activate resting NK cell degranulation (*SI Appendix*, Fig. S3 *A* and *B*). Thus, activation of resting KIR2DS4^+^ NK cells does not require synergistic stimulation of multiple receptors.

**Fig. 3.**
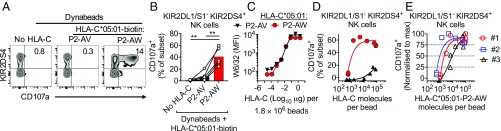
Peptide/HLA-C is sufficient to activate KIR2DS4^+^ NK cells. (*A*) Flow cytometry biplots displaying CD107a expression on CD56^dim^ KIR2DL1/S1^−^ NK cells after stimulation with unconjugated Dynabeads and Dynabeads conjugated to biotinylated HLA-C*05:01 refolded with P2-AV or P2-AW. Expression of KIR2DS4 is shown on the *y* axis. (*B*) Expression of CD107a on CD56^dim^ KIR2DL1/S1^−^ KIR2DS4^+^ NK cells after stimulation by unconjugated Dynabeads and Dynabeads conjugated to biotinylated HLA-C*05:01 refolded with P2-AV or P2-AW. Data with NK cells from five donors are shown. (*C*) Binding of W6/32 mAb to Dynabeads conjugated to HLA-C*05:01 biotin refolded with P2-AV or P2-AW. Two micrograms of HLA-C were diluted fivefold eight times in PBS (final volume = 20 μl) and each dilution conjugated to 1.8 million dynabeads. One of three independent experiments is shown. (*D*) Expression of CD107a on CD56^dim^ KIR2DL1/S1^−^ KIR2DS4^+^ NK cells after stimulation with Dynabeads bearing increasing densities of biotinylated HLA-C*05:01 refolded with P2-AV or P2-AW. Antigen density was calculated by quantitative flow cytometry. Data from donor 2 are shown. (*E*) Expression of CD107a on CD56^dim^ KIR2DL1/S1^−^ KIR2DS4^+^ NK cells after stimulation with Dynabeads bearing increasing antigen densities of biotinylated HLA-C*05:01 refolded with P2-AW. Data from three donors were normalized to the maximum value of CD107a^+^ for each donor. Maximum responses were for donor 1 (42%), donor 2 (65%), and donor 3 (32%). Statistical significance was assessed by one-way ANOVA with Tukey’s multiple comparisons test (**P < 0.01).

To test whether HLA-C is sufficient to activate KIR2DS4^+^ NK cells, biotinylated HLA-C*05:01 refolded with P2-AV or P2-AW was conjugated to streptavidin Dynabeads. These beads stained strongly with a β_2_M-specific mAb (*SI Appendix*, Fig. S3*C*). KIR2DS4^+^KIR2DL1/S1^−^ (S4^+^L1/S1^−^) NK cells degranulated strongly in response to beads conjugated to HLA-C*05:01 refolded with P2-AW, but not with P2-AV, while KIR2DS4^−^ NK cells were not stimulated by any of the beads (Fig. 3 *A* and *B*). Activation of KIR2DS4^+^ cells was dependent on bead number, and expression of KIR2DL1/S1 on KIR2DS4^+^ NK cells inhibited NK cell activation (*SI Appendix*, Fig. S3 *D* and *E*), consistent with our previous results with peptide-loaded 221-C*05:01-ICP47 cells (Fig. 2*H*). Next, HLA-C*05:01 conjugation to Dynabeads was titrated such that the number of HLA-C molecules per bead ranged from 0.5 × 10^3^ to 0.5 × 10^5^ (Fig. 3*C*). The number of HLA-C molecules per bead was estimated using quantitative flow cytometry, by generating a standard curve from beads with known antigen densities (*SI Appendix*, Fig. S3*F*). S4^+^L1/S1^−^ NK cells showed a sharp activation threshold, with half-maximal responses at 2,000 and 1,000 HLA-C molecules per bead for donors 1 and 2, respectively (Fig. 3*E*). In contrast, S4^+^L1/S1^−^ NK cells were weakly stimulated with beads conjugated with HLA-C*05:01-P2-AV at antigen densities greater than 20,000 HLA-C molecules per bead (Fig. 3*D* and *SI Appendix*, Fig. S3*G*). NK cells from a third donor showed a weaker response, with a gradual increase in the activation of S4^+^L1/S1^−^ NK cells with increasing HLA-C antigen density and a half-maximal response at 8,000 HLA-C molecules per bead (Fig. 3*E*). We concluded that HLA-C alone, immobilized to beads, is sufficient to activate KIR2DS4^+^ NK cells across a range of antigen densities in the absence of ligands for other coactivation receptors or adhesion molecules.

### Functional Presentation of Endogenous P2-AW Peptide by HLA-C*05:01 to KIR2DS4^+^ NK Cells.

**Fig. 4.**
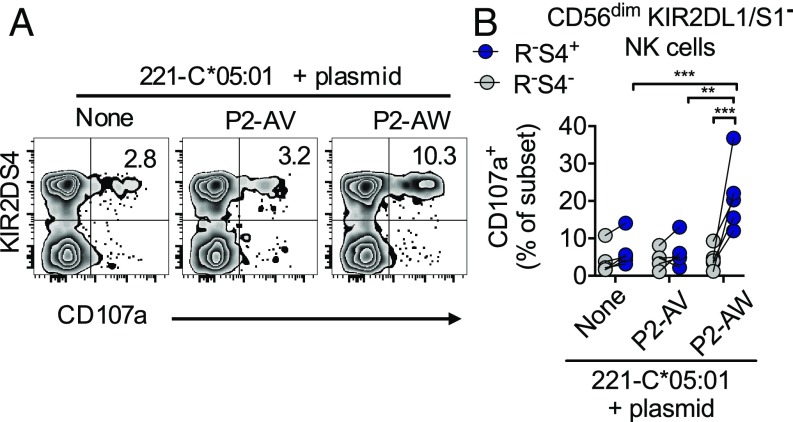
Functional presentation of endogenous P2-AW peptide by HLA-C*05:01 to KIR2DS4^+^ NK cells. (*A*) Expression of CD107a on KIR2DS4^+^ and KIR2DS4^−^ NK cell subsets in response to 221-C*05:01 cells transduced with plasmids encoding P2-AV or P2-AW. NK cells were gated as CD56^dim^ KIR2DL1/S1^−^R^−^ as in Fig. 2*A*. One representative experiment of five is shown. (*B*) Expression of CD107a on NK cell subsets as in *A* in response to 221-C*05:01 cells transduced with plasmids encoding P2-AV or P2-AW. Data from five independent experiments are shown. Statistical significance was assessed by two-way ANOVA with Sidak’s multiple comparisons test (**P < 0.01, ***P < 0.001).

Our experiments thus far demonstrated that KIR2DS4^+^ NK cells can recognize peptides presented by HLA-C*05:01 in a highly selective manner. For these experiments, the peptides presented by HLA-C*05:01 have been dominated by single peptide sequences, in the form of recombinant HLA-C refolded with peptide or TAP-deficient cells loaded with peptide. While these are useful experimental tools, under physiological conditions, HLA-C*05:01 does not present single peptides, but many peptides of different sequences (10, 30, 31). To test whether KIR2DS4^+^ NK cells could recognize peptide in TAP-sufficient cells where HLA-C*05:01 presents many different peptides, we expressed the peptide P2-AW or P2-AV in 221-C*05:01 cells using a retrovirus (32) (Fig. 4*A* and *SI Appendix*, Fig. S5*A*). Transduced cells were marked by expression of mCherry, and after drug selection, both minigenes were expressed in over 90% of cells (*SI Appendix*, Fig. S5*B*). In degranulation assays, R^−^S4^+^ NK cells responded weakly to both 221-C*05:01 cells and those transduced with P2-AV (Fig. 4*B*). In contrast, R^−^S4^+^ NK cells exhibited enhanced degranulation in response to 221-C*05:01 cells expressing the P2-AW peptide (Fig. 4*B*). This activation was KIR2DS4-specific, as R^−^S4^−^ NK cells exhibited low responses to both 221-C*05:01 cells and those transduced with P2-AV or P2-AW. Thus, KIR2DS4 can detect a stimulatory peptide presented by HLA-C*05:01 in TAP-sufficient cells that present many different peptide sequences.

### A “Self” Peptide Eluted from HLA-C*05:01 Carrying Trp at P8 Is a KIR2DS4 Epitope.

We reasoned that peptides containing Trp at p8 may be enriched for KIR2DS4 binding peptides as, in the context of P2-AY, Trp at p8 conferred stronger binding of KIR2DS4 to HLA-C*05:01 than Tyr or Phe (Fig. 1). Peptides eluted from HLA-C*05:01 contain a low frequency of Trp (*SI Appendix*, Fig. S5), which was similar to peptides eluted from other HLA-C allotypes (30, 31). Trp is rare at p8 for all HLA-C allotypes, ranging from 0–2.6%, and is found at a frequency of 0.6% in HLA-C*05:01 peptides (*SI Appendix*, Fig. S5*B*). The scarcity of peptides containing Trp at p8 presented by HLA-C*05:01 may explain the lack of KIR2DS4-Fc binding to 221-C*05:01 cells.

**Fig. 5.**
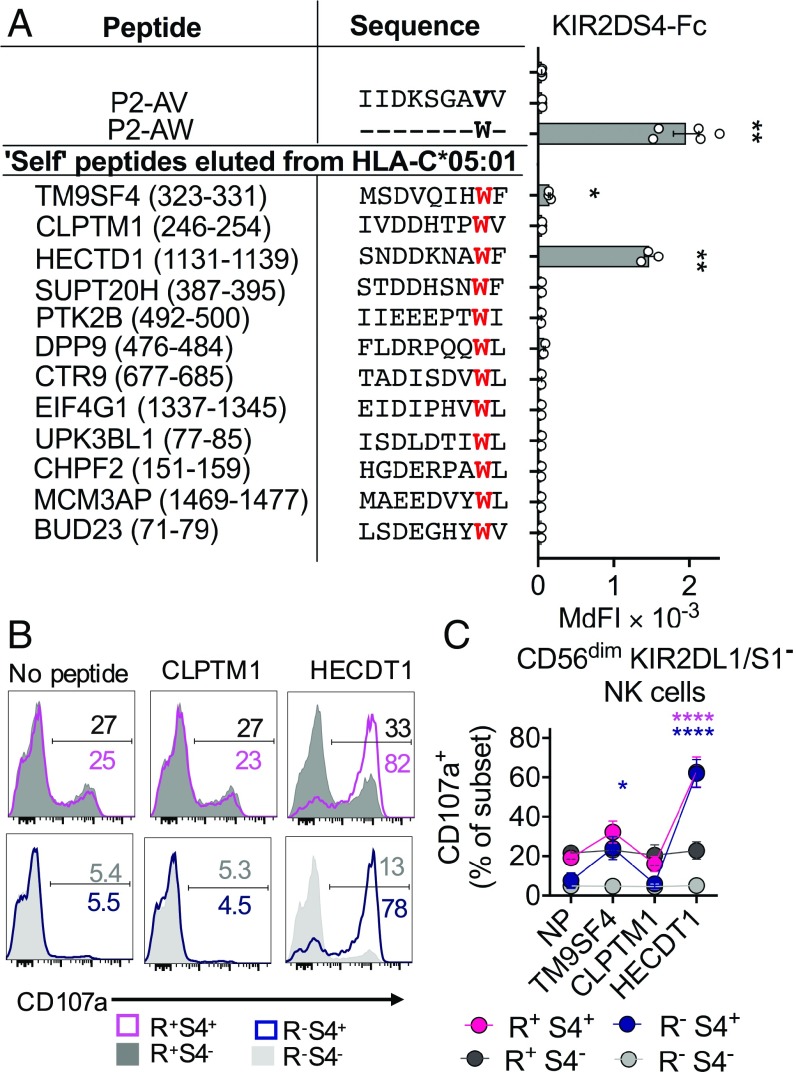
“Self” peptide eluted from HLA-C*05:01 with Trp at p8 is a KIR2DS4 epitope. (*A*) KIRD2S4-Fc binding to 221-C*05:01-ICP47 cells loaded with each one of 12 endogenous “self” peptides carrying Trp at p8, compared with P2-AV, P2-AW, or no peptide. The 12 “self” peptides were identified from two studies, which eluted and sequenced a total of 1,674 unique 9-mer peptides from purified HLA-C*05:01. TM9SF4_323–311_ and HECTD1_1131–1139_ were identified in both studies. The amino acid sequence of each “self” peptide and position within the protein of origin is listed. MdFI, median fluorescence intensity. (*B*) Flow cytometry histograms displaying CD107a expression on CD56^dim^ KIR2DL1/S1^−^ NK cell subsets in response to 221-C*05:01-ICP47 cells with loaded with “self” peptides from proteins CLPTM1 and HECDT1 or no peptide. CD56^dim^ KIR2DL1/S1^−^ NK cells were gated as in Fig. 2*A*. (*C*) Expression of CD107a on NK cell subsets as in *B*. Mean and SEM from three independent experiments with NK cells from different donors are shown. Statistical significance was assessed by one-way ANOVA (*A*) with Tukey’s multiple comparisons test and two-way ANOVA (*C*) with Dunnett’s multiple comparisons test (*P < 0.05, **P < 0.01, ****P < 0.0001). Significance is indicated in comparison to no peptide (NP) and is color-coded by NK cell subsets.

We synthesized and tested the 12 peptides with Trp at p8 that had been eluted from HLA-C*05:01 for KIR2DS4 binding and activation of KIR2DS4^+^ NK cells. One strong KIR2DS4 binding “self” peptide and one weak KIR2DS4 binding “self” peptide were identified (Fig. 5). The KIR2DS4 weak binding peptide was TM9SF4_323–311_ (MSDVQIHWF). TM9SF4 is a member of the transmembrane superfamily 9, a highly conserved family across evolution with roles in cell adhesion, phagocytosis, and autophagy (33). The KIR2DS4 strong binding “self” peptide was HECTD1_1131–1139_ (SNDDKNAWF). The homologous to the E6-AP carboxyl terminus (HECT) domain E3 ubiquitin ligase 1 (HECTD1) has been linked to cholesterol export from macrophages (34) and suppression of epithelial-to-mesenchymal transition in cancer metastasis (35). Both TM9SF4 and HECTD1 are expressed in all tissues at the RNA and protein levels (36). That only two of 12 peptides with Trp at p8 bound KIR2DS4 emphasized the high peptide specificity of KIR2DS4 binding to HLA-C*05:01 and that Trp is not sufficient to identify KIR2DS4 binding peptides.

### A Conserved KIR2DS4 Epitope Derived from Recombinase A Is Shared by Hundreds of Species of Bacteria.

**Fig. 6.**
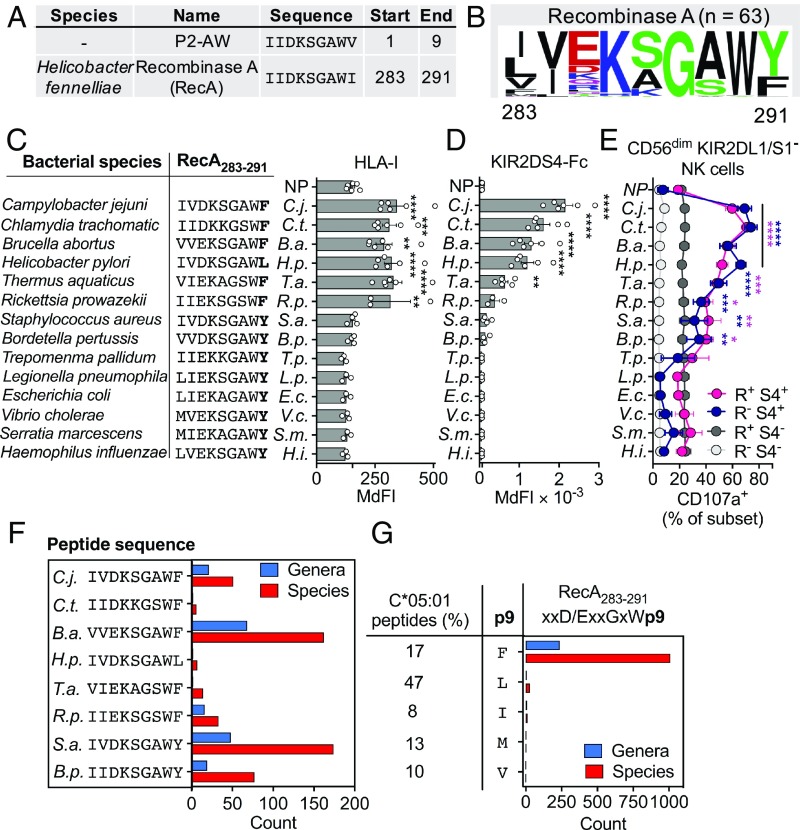
KIR2DS4 epitope in a conserved RecA sequence shared by hundreds of bacterial species. (*A*) Amino acid sequence alignment of peptide P2-AW and a RecA_283–291_ sequence from *H. fennelliae*. (*B*) Peptide sequence motif present in RecA_283–291_ sequences from 63 species of bacteria. (*C*) Stabilization of HLA-I expression on 221-C*05:01-ICP47 cells incubated overnight at 26 °C with 100 μM 14 different RecA_283–291_ peptides. RecA_283–291_ peptide sequences are from 14 species of bacteria. Data are shown as median fluorescence intensity (MdFI) (*n* = 3–5). (*D*) KIR2DS4-Fc binding to 221-C*05:01-ICP47 cells incubated with peptides as shown in *C*. Data are shown as MdFI (*n* = 3–5). (*E*) Expression of CD107a on CD56^dim^ NK cell subsets in response to 221-C*05:01-ICP47 cells loaded with peptides as in *C*. CD56^dim^ KIR2DL1/S1^−^ NK cells were gated as in Fig. 2*A*. Data are mean and SEM from three independent experiments with NK cells from different donors. (*F*) Number of genera and species of bacteria with RecA_283–291_ peptide sequences identical to those that activate KIR2DS4^+^ NK cells as in *F*. (*G*) Number of genera and species of bacteria with RecA_283–291_ peptide sequences with the motif xxD/ExxGxWp9 (x = any residue, p9 = C-terminal residue). The frequency of F, L, I, M, and V at the C terminus of 9-mer peptides eluted from HLA-C*05:01 is also shown. Statistical significance was assessed by one-way (*D* and *E*) and two-way (*F*) ANOVA (*P < 0.05, **P < 0.01, ***P < 0.001, ****P < 0.0001). Significance is indicated in comparison to no peptide, and in *F* is color-coded by NK cell subsets.

To explore whether KIR2DS4 recognizes pathogen-derived peptides, we searched the proteomes of prokaryotes for sequences similar to P2-AW. P2-AW (IIDKSGAWV) showed remarkable homology to residues 283–291 of recombinase A (RecA) of *Helicobacter fennelliae* (IIDKSGAWI; Fig. 6*A*). RecA is the prototypical DNA recombinase and is essential for DNA damage repair by homologous recombination (37). A previous study aligned the sequences of RecA proteins from 63 species of bacteria and found amino acid sequence similarity ranged from 43–100% (38). Residues 283–291 are highly conserved, and G_288_, in particular, is conserved in all 63 species; W_290_ is found in 61 of 63 species; and acidic residues at p285 are found in 40 of 63 species (*SI Appendix*, Fig. S6*A*). A logo-motif for RecA_283–291_ from these 63 species is shown in Fig. 6*B*.

We tested 14 RecA_283–291_ peptides with different sequences, each containing Asp or Glu at p3, a critical anchor residue for HLA-C*05:01. Due to the high level of conservation, these 14 peptides cover more than 14 species of bacteria; for example, RecA_283–291_ from *Yersinia pestis* is identical to that of *Escherichia coli*. RecA_283–291_ peptides with Phe or Leu at the C terminus stabilized HLA-C*05:01 well, bound KIR2DS4, and activated KIR2DS4^+^ NK cells (Fig. 6 *C*–*F*). Epitopes derived from the pathogens *Chlamydia trachomatis*, *Campylobacter jejuni*, *Brucella abortus*, and *Helicobacter pylori* bound KIR2DS4 the strongest and conferred potent stimulation of KIR2DS4^+^ NK cells (Fig. 6 *C*–*F*). The epitopes from *Thermus aquaticus* and *Rickettsia prowazekii* conferred an intermediate level of KIR2DS4 binding, while epitopes from *Staphylococcus aureus* and *Bordetella pertussis* conferred weak KIR2DS4 binding (Fig. 6 *C*–*F*). Of the 14 peptides, those with Tyr at the C terminus were poor ligands for HLA-C*05:01 and conferred low HLA-I stabilization and little or no KIR2DS4 binding (Fig. 6 *C*–*F*). This is consistent with the very low frequency of peptides eluted from HLA-C*05:01 that use Tyr as a C-terminal anchor (*SI Appendix*, Fig. S6*B*).

To evaluate how many species of bacteria may contain KIR2DS4 epitopes, we downloaded all bacterial RecA sequences from the National Center for Biotechnology Information Protein database (https://www.ncbi.nlm.nih.gov/protein). We identified over 100 bacterial species with RecA_283–291_ sequences identical to those that bound KIR2DS4 (Fig. 6*F*). Of the four sequences that bound KIR2DS4 the strongest, those identical to the *B. abortus* sequence were the most frequent. This included the sequence from *Brucella melitensis*, another pathogenic species of the *Brucella* genus (39). Taking a broader approach, we generated a RecA_283–291_ motif (xxD/ExxGxWp9) accounting for the essential role of acidic resides at p3 for binding HLA-C*05:01, the high conservation of p6 G, and the importance of p8 Trp for binding KIR2DS4 (Fig. 6*G*). We allowed for any amino acid at all other positions (x) except p9. To focus our analysis on peptides predicted to be presented well by HLA-C*05:01, we counted only RecA_283–291_ sequences where Phe, Leu, Ile, Met, or Val was at p9, all common C-terminal anchors for HLA-C*05:01 peptides (*SI Appendix*, Fig. S6*B*). Using this motif, we identified over 1,000 different bacterial species that contain RecA_283–291_ sequences that have the potential to be presented by HLA-C*05:01 and bind KIR2DS4 (Fig. 6*G*). The majority of species containing RecA_283–291_ sequences predicted to bind KIR2DS4 were from Proteobacteria, and within the Proteobacteria, the most common order was Rhizobiales (*SI Appendix*, Fig. S6 *C* and *D*). All bacterial species containing RecA sequences predicted to be presented by HLA-C*05:01 and recognized by KIR2DS4 are shown in Dataset S1. Together, our data suggest the possibility that HLA-C*05:01^+^ individuals expressing KIR2DS4 may have an evolutionary advantage in bacterial immunity, through the ability of their NK cells to recognize RecA epitopes presented by HLA-C*05:01.

### Allele Frequency of KIR2DS4-fl Is Inversely Correlated with the Frequency of HLA-C*05:01.

**Fig. 7.**
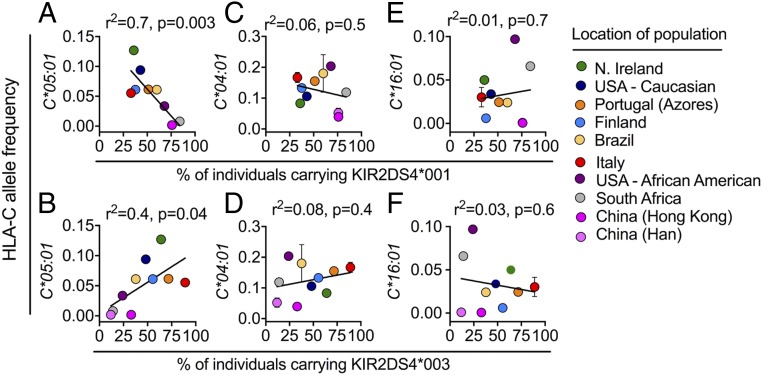
Functional KIR2DS4 allele is inversely correlated with HLA-C*05:01. Allele frequency of HLA-C*05:01 (*A* and *B*), HLA-C*04:01 (*C* and *D*), and HLA-C*16:01 (*E* and *F*) correlated with the percentage of individuals who are KIR2DS4*001^+^ (*A*, *C*, and *E*) and KIR2DS4*003^*+*^ (*B*, *D*, and *F*) in 10 populations. Data are from the Allele Frequency Net Database (www.allelefrequencies.net).

The allele frequency of functional KIR2DS4-fl (KIR2DS4*001) is positively correlated with HLA-A*11, a previously identified KIR2DS4 ligand (22). We therefore examined the allele frequency of HLA-C*05:01 in 10 populations where the percentage of individuals carrying KIR2DS4-fl (KIR2DS4*001) and KIR2DS4-del (KIR2DS4*003) is known (http://www.allelefrequencies.net/) (40). There was a strong inverse correlation (*r* = −0.83, *r*^2^ = 0.7, *P* = 0.003) with the allele frequency of HLA-C*05:01 and the percentage of individuals carrying KIR2DS4*001 (Fig. 7*A*). Conversely, there was a positive correlation (*r* = 0.64, *r*^2^ = 0.4, *P* = 0.04) with the allele frequency of HLA-C*05:01 and the percentage of individuals carrying KIR2DS4*003 (Fig. 7*B*). The highest frequency of HLA-C*05:01 is found in those with European Caucasian ancestry represented by two populations: one from Northern Ireland (0.13) and the other from the United States (0.09) (Fig. 7*A*). The percentage of individuals carrying KIR2DS4*001 in these two populations was lower than 45% (Fig. 7*A*). In contrast, populations with a very low frequency of HLA-C*05:01, shown here by two Chinese populations (0.0018) and a South African population (0.008), had greater than 75% of individuals carrying KIR2DS4*001 (Fig. 7*A*). We saw similar results using data from nine populations with high-resolution KIR allele typing, where the allele frequencies of all three genotypes, KIR2DS4-fl, KIR2DS4-del, and KIR2DS4^−^, were known (*SI Appendix*, Fig. S7 *A* and *B*). The allele frequency of HLA-C*05:01 was inversely correlated with the frequency of KIR2DS4-fl alleles (*r* = −0.85, *r*^2^ = 0.7, *P* = 0.004), and positively correlated with the frequency of KIR2DS4-del alleles (*r* = 0.95, *r*^2^ = 0.9, *P* = 0.0004) and the frequency of the KIR2DS4^−^ genotype combined with the frequency of KIR2DS4-del alleles (*r* = 0.85, *r*^2^ = 0.7, *P* = 0.004; *SI Appendix*, Fig. S7 *A* and *B*). This effect appeared to be unique to HLA-C*05:01, as the allele frequencies of the other KIR2DS4 ligands, HLA-C*04:01 and HLA-C*16:01 (22), showed no correlation with the percentage of individuals carrying KIR2DS4*001 or KIR2DS4*003 (Fig. 7 *C*–*F*). Therefore, populations with a higher frequency of HLA-C*05:01 have lower frequencies of the functional receptor KIR2DS4-fl.

## Discussion

The concept of an innate receptor with specificity for one form of a highly variable antigen, such as an MHC/peptide complex, is counterintuitive. Yet, members of the KIR family bind to HLA-I allotypes with various degrees of selectivity for peptides (10, 12, 41). In the case of the activating members of the KIR family, for which ligands are less well defined, a reasonable hypothesis was that they serve to recognize conserved features of modified “self.” Here, we describe an extreme case of peptide selectivity of an activating KIR, which is restricted by HLA-C*05:01. Peptides that promote binding of KIR2DS4 carried a Trp at p8 of 9-mer peptides. Recognition of this peptide/HLA-C*05:01 complex by KIR2DS4^+^ NK cells induced potent activation of NK cells, and could even activate unlicensed NK cells. Interestingly, optimal peptides for activation of KIR2DS4^+^ NK cells identified in this study included an epitope that is highly conserved in the essential protein RecA expressed by hundreds of bacterial species, suggesting KIR2DS4 may have evolved to contribute to bacterial innate immunity.

RecA is essential for repair of damaged DNA by homologous recombination and is critical for the DNA damage response in *E. coli* (37). The epitope in the bacterial protein RecA for KIR2DS4 binding contains Trp at p8, and this position (residue 290 in *E. coli*) is conserved across bacteria, including many human pathogens. Trp is a rare amino acid in general (1.3% of the human proteome), which is reflected in its low representation in peptides eluted from HLA-C (30, 31). Only 0.6% of peptides (*n* = 2,035) eluted from HLA-C*05:01 contained Trp at p8. Of 12 such peptides, only one bound KIR2DS4, providing an explanation for the lack of KIR2DS4 binding to, or activation of KIR2DS4^+^ NK cells by, 221-C*05:01 cells. The low abundance of peptides, including Trp at p8, provides the opportunity to detect similar but more abundant peptides, perhaps modified peptides or peptides derived from pathogens. We propose that KIR2DS4 is not specific for nonself but for “rare self,” which provides the opportunity to detect abundant foreign peptides carrying a “rare self” epitope.

The major function of inhibitory KIRs is recognition of “self” to inhibit NK cell activation and dictate NK cell licensing (2). Inhibitory KIRs exhibit limited peptide selectivity and bind HLA-I in the context of many biochemically diverse peptides. However, differences in peptide selectivity exist between KIRs. Recently, we compared KIR2DL1 binding with HLA-C*05:01 and KIR2DL2/3 binding with HLA-C*08:02 in the presence of the same peptides. HLA-C*05:01 and HLA-C*08:02 differ only by the two amino acids that define the C1 and C2 epitopes and could be loaded with the same peptides. While KIR2DL1 bound HLA-C*05:01 in the presence of 24 of 28 peptides, KIR2DL2 and KIR2DL3 bound HLA-C*08:02 in the presence of only 13 and 11 of the same peptides, respectively (10). P44 of KIR2DL1 (Met) and KIR2DL2/3 (Lys) dominates in determining KIR specificity for C2 and C1 allotypes, respectively (9, 11). However, Lys44 receptors exhibit cross-reactive binding to C2 allotypes (42, 43). KIR2DS4, which carries Lys44, has the capacity to bind a small subset of C1 and C2 allotypes. The high peptide selectivity of KIR2DS4 is reminiscent of cross-reactive KIR binding, where KIR2DL2/3 bound HLA-C*05:01 and KIR2DL1 bound HLA-C*08:02 in the presence of five and two of 28 of the same peptides, respectively (10). KIR2DS4 was even more peptide-selective than this, binding none of the same 28 peptides previously studied and only in the presence of the few peptides described here.

Examples of pathogenic bacteria that carry an epitope within RecA for HLA-C*05:01–restricted binding of KIR2DS4 include the following: *H. pylori*, an infectious agent associated with peptic ulcers and gastric cancers (44); bacteria of the *Brucella* genus, which cause a zoonotic infection called brucellosis (45); *C. jejuni*, a major cause of gastroenteritis with an incidence as common as that caused by *Salmonella* infections (46); and *C. trachomatis*, which is the most common sexually transmitted infection, with over 90 million cases annually worldwide (47). There are bacterial species with a RecA sequence that lacks an Asp or Glu at p3, which is required for peptide binding to HLA-C*05:01 (10, 30, 31). Such species include *Mycobacterium tuberculosis*, which carries the conserved Trp at p8 but has an Arg at p3 (LIRKSGAWF). Additionally, other species such as *E. coli* and *Y. pestis* carry a Tyr at p9, which is a poor C-terminal anchor for HLA-C*05:01. It is possible that other HLA-C allotypes may also present these RecA peptides by accommodating other amino acids at p3 or p9, expanding the coverage of pathogenic bacteria detected by KIR2DS4^+^ NK cells.

KIR2DS4 is a strong activator of NK cells and was sufficient on its own to elicit functional responses by KIR2DS4^+^ NK cells. Using an assay with recombinant HLA-C*05:01 carrying an optimal Trp at p8 peptide conjugated to beads, we determined the number of HLA-C molecules required for half-maximal activation of KIR2DS4^+^ NK cells to be from 1,000 to 8,000, suggesting the number of molecules required to activate KIR2DS4^+^ NK cells may be within a physiological range. Furthermore, the presence of ligands on target cells for adhesion molecules and coactivation receptors on NK cells may reduce the number of HLA-C/peptide complexes on human cells required to activate KIR2DS4^+^ NK cells. As a tool to determine whether an endogenous peptide carrying an epitope for HLA-C*05:01–restricted binding of KIR2DS4 could be presented at the plasma membrane and detected by KIR2DS4^+^ NK cells, we expressed peptide P2-AW with a retrovirus. KIR2DS4^+^ NK cells were activated by 221-C*05:01 cells only after endogenous expression of the right peptide. Thus, KIR2DS4^+^ NK cells can detect antigen presented by TAP-sufficient cells, presumably in the context of many other peptides that do not bind KIR2DS4. Although the number or the proportion of HLA-C molecules that present the RecA peptide epitope in our expression system is unknown, our estimate of the number of peptide/HLA-C complexes bound to beads required for stimulation is significantly lower than the reported estimate of ∼10^5^ HLA-C molecules required to inhibit NK cells through KIR2DL1 (48). The strong activation signals transmitted by KIR2DS4 overcome the lack of licensing but were still controlled through coengagement of inhibitory KIR2DL1 on NK cells. NK cell activation by KIR2DS4 must occur in scenarios where HLA-C*05:01 is expressed, unlike “missing self” NK responses due to MHC-I down-regulation. It is likely that KIR2DS4^+^ NK cell subsets that coexpress inhibitory receptors will be inhibited, depending on the donor KIR and HLA-I genotypes. However, we demonstrated that even those KIR2DS4^+^ NK cells that lack inhibitory receptors (R^−^S4^+^) can be potently activated via KIR2DS4. Under such scenarios, we propose that this subset (R^−^S4^+^) would be most potently activated.

As NK cells are potently activated by KIR2DS4, overstimulation could have negative consequences, such as inflammation and autoimmunity. The negative correlation between the frequency of HLA-C*05:01 and KIR2DS4-fl alleles in different geographically defined populations suggests there are detrimental consequences for populations with high frequencies of this receptor and ligand pair, indicative of balancing selection. It demonstrates a genetic interaction between them and suggests that the KIR2DS4-del alleles may have originated in populations with a high frequency of HLA-C*05:01. The highest frequency of the HLA-C*05:01 allele is in European populations, including Northern Ireland, where the KIR2DS4-del allele was first described (21, 40, 49). This is evidence for balancing selection between an activating KIR and its ligand, which is reminiscent of examples of balancing selection in populations with high frequencies of inhibitory KIR ligands (50). For example, the frequency of C2 allotypes is very high in the KhoeSan tribes of southern Africa, where novel KIR2DL1 alleles were discovered that have lost C2 specificity or lost the capacity to signal (51). Similarly, in the Yucpa tribe of South America, where C1 frequency is highest worldwide, novel KIR2DL3 alleles were discovered that encode receptors with weaker avidity for C1 allotypes (52).

Activating KIRs are rapidly evolving and differ even among higher primate species (53). Activating KIRs evolved from their inhibitory counterparts and coopted existing evolutionarily conserved signaling adaptor molecules like DAP12 (53). However, there is no ancestral lineage of activating KIR genes and they appear to undergo periods of positive selection followed by negative selection. As such, many activating KIRs, including *KIR2DS4*, are not fixed on either KIR A or B haplotypes, suggesting they are under current selection. Beneficial effects of a novel activating KIR could include resistance to pathogens and improved reproductive success, while negative effects could include autoimmunity and too high a birth weight (53). Human pregnancy is a physiological process impacted by KIR genes, including KIR2DS4 (18, 19). Human birth weight is under balancing selection as babies born too big or too small are less likely to survive, and combinations of KIRs and their ligands associate with both ends of this spectrum (18, 54). Given that KIR2DS4 protects from preeclampsia, a disorder of insufficient blood supply to the fetus, it is possible that a high frequency of KIR2DS4 and HLA-C*05:01 had a negative effect in those populations because of too high a birth weight. This kind of balancing effect has been observed for KIR2DS1 and HLA-C2, where this combination of C2 and an activating receptor is associated with increased birth weight, while the combination of C2 and an inhibitory receptor is associated with low birth weight and increased risk of preeclampsia (18, 54).

An NK cell-activating receptor with high selectivity for pathogen-derived peptides is not without precedent. The CD94:NKG2C receptor was recently shown to recognize peptides derived from the UL40 protein of human cytomegalovirus (HCMV), presented by HLA-E (55). Recognition of these peptides is thought to drive the formation of adaptive NK cells in HCMV-infected individuals (55). Furthermore, a recent study discovered that KIR2DS2, another activating KIR, recognizes HLA-C–bound peptides derived from a conserved region of the NS3 helicase of the Flavivirus family (56). This family includes several pathogens such as hepatitis C, Ebola, Zika, and West Nile viruses. It is likely that KIR2DS2^+^ and CD94:NKG2C^+^ NK cells participate in immune defense through direct recognition of virus-infected cells. Here, we demonstrated that KIR2DS4 is a bona fide HLA-C binding receptor that may have evolved to play a protective role in immune defense against bacteria. In the context of bacterial infections, the stimulation of IFN-γ production by KIR2DS4^+^ NK cells is likely to contribute to clearance of bacterial pathogens. Phagocytic, antigen-presenting cells (APCs) such as monocytes, macrophages, and dendritic cells (DCs) are the first line of defense against invading bacteria. NK cells cooperate with these cell types to produce IFN-γ via cell contact-dependent mechanisms and IL-12 production (57–59). It is possible that KIR2DS4^+^ NK cells could directly recognize bacterially infected cells; indeed, *C. trachomatis*, *C. jejuni*, and *B. abortus* are intracellular bacteria (45, 47, 60). An additional scenario is that KIR2DS4^+^ NK cells could be activated upon interaction with APCs that present RecA epitopes on HLA-C to produce IFN-γ early during infection, and to facilitate a T helper 1 response necessary to clear the bacterial infection. In this case, the RecA epitope would be presented by HLA-C through cross-presentation, the process whereby exogenous antigens enter the MHC-I pathway (61). The DC subset associated with efficient cross-presentation consists of classical DC1 (cDC1) (62). NK cells interact with DCs, and the interplay between cDC1 and NK cells was shown recently to be important for antitumor responses and associated with greater responses to checkpoint blockade therapy (63, 64). Notably, NK cells produced CCL5 and XCL1, which facilitated cDC1 recruitment to the tumor site (63). Thus, in addition to IFN-γ, NK cells may contribute to enhanced bacterial clearance through promotion of cDC and T cell interactions. Disease association studies of KIR2DS4 and HLA-C*05:01 with outcome of bacterial infections will be needed to test this idea further.

## Methods

### Cell Lines.

In this study, 221 cells and 221 cells expressing HLA-C*04:01 were used (provided by J. Gumperz and P. Parham, Stanford University, Stanford, CA). The 221 cells expressing HLA-C*05:01 (221-C*05:01) and those expressing the TAP inhibitor ICP47 (221-C*05:01-ICP47) were previously described (10). All 221 cells were cultured in Iscove’s modified Dulbecco’s medium (Gibco) supplemented with 10% fetal calf serum (FCS).

### Peptide HLA-C Stabilization Assays.

Peptide stabilization of HLA-C was assessed by flow cytometry largely as described (10). A total of 10^5^ cells were incubated overnight at 26 °C with 100 μM synthetic peptide. The following day, cells were stained with APC HLA-I mAb (W6/32; Biolegend) at 4 °C. Peptides were synthesized by Genscript.

### KIR-Fc Binding Assay.

KIR-Fc binding to cell lines and peptide-loaded cells was assessed by flow cytometry largely as described (10). KIR2DL1*001-Fc and KIR2DS4*001-Fc (1844-KR and 1847-KR; R&D Systems) were conjugated to protein-A Alexa Fluor 647 (Invitrogen) by overnight incubation at a ratio of 9:1 (molar) at 4 °C and then diluted to 3.6 μg/mL (KIR2DL1*001-Fc) and 9 μg/mL (KIR2DS4*001-Fc) in phosphate-buffered saline (PBS) + 2% FCS. A total of 10^5^ cells were placed in 96 flat-well plates, resuspended in 25 μL of KIR-Fc, and incubated at 4 °C for 1 h. For peptide-loaded cells, KIR-Fc binding was assessed after overnight incubation of cells at 26 °C with 100 μM synthetic peptide. KIR-Fc binding to 221 cells or protein A-Alexa Fluor 647 alone was used to determine baseline values for KIR-Fc binding. Cells were washed with PBS three times, and data were acquired by flow cytometry.

### NK Cell Functional Assays.

NK cell degranulation assays were carried out largely as described (10). Primary, resting NK cells were isolated by negative selection from peripheral blood mononuclear cells and were greater than 95% CD56^+^ and less than 5% CD3^+^ (EasySep NK Cell Isolation Kit; STEMCELL Technologies). Donors were screened for the presence of KIR2DS4 with the mAb JJC11.6 (Miltenyi Biotec). A total of 10^5^ resting NK cells were mixed at a ratio of 1:1 with 221 cell lines or peptide-loaded target cells in presence of 1 μL of BV421 anti-CD107a mAb for 2 h at 37 °C (H4A3; Biolegend 328626). NK cells were then stained with mAbs to identify subsets based on the expression of KIR2DL1/S1 (Beckman Coulter; EB6, APC), receptor [R; Beckman Coulter; NKG2A;Z199, KIR2DL2/L2/S2;GL183, CD158e1/2;Z27, all phycoerythrin (PE) conjugated], and KIR2DS4 (S4; Miltenyi Biotec, JJC11.6, PE-Vio770). For intracellular cytokine staining, NK cells were mixed with targets for 6 h at 37 °C, and Golgi Plug (BD Biosciences, 555029) was added after 1 h. Cells were then fixed and permeabilized with Cytofix/Cytoperm (BD Biosciences, 554714) and stained for IFN-γ (BD Biosciences; B27, 562988, BV421) and TNF-α (BD Biosciences; MAb11, 554512, fluorescein isothiocyanate). Cells were washed with PBS three times, and data were acquired by flow cytometry. To identify KIR2DS1^−^ donors, NK cells were stained with mAbs to KIR2DL1 (R&D Systems; 143211, PE) and KIR2DL1/S1 (Beckman Coulter; EB6, APC) as described (65). For experiments where NK cells were mixed with beads, 10^5^ resting NK cells were mixed with 1.8 × 10^6^ streptavidin M280 Dynabeads in V-bottomed 96-well plates in the presence of 1 μL of BV421 anti-CD107a mAb for 2 h at 37 °C (H4A3; Biolegend, 328626). Streptavidin M280 Dynabeads were conjugated to biotinylated HLA-C*05:01 refolded with P2-AV or P2-AW as described below.

### Quantification of HLA-C Conjugation to Beads.

Biotinylated recombinant HLA-C*05:01 refolded with P2-AV or P2-AW was supplied by the NIH Tetramer Core Facility. Biotinylated HLA-C*05:01 refolded with P2-AV or P2-AW (2 μg) was conjugated overnight at 4 °C with 6 × 10^6^ streptavidin M280 Dynabeads in 20 μl PBS. Seven fivefold dilutions of HLA-C were also conjugated in the same way. Dynabeads were washed five times with PBS and resuspended in 100 μL of PBS for use as targets in NK cell degranulation assays or flow cytometry staining. The density of HLA-C per bead was calculated using QIFIKIT calibration beads according to the manufacturer’s instructions (Dako, K0078). Calibration beads contained five populations of beads conjugated to different numbers of mouse Ig. Calibration beads were stained with F(ab′)_2_ FITC-conjugated goat anti-mouse immunoglobulins (Dako, F0479) at a 1:50 dilution for 1 h at 4 °C. Beads were washed three times with PBS and analyzed by flow cytometry. The mean fluorescence intensity (MFI) for each bead population was correlated with the number of conjugated mouse immunoglobulins, provided by the manufacture (lot 20050787), to generate a standard curve. Ten microliters (6 × 10^5^) of HLA-C–conjugated Dynabeads were stained with anti–HLA-I mAb W6/32 (1 mg/mL; Acites) for 1 h and then washed with PBS three times. The Dynabeads were then stained with F(ab′)_2_ FITC-conjugated goat anti-mouse immunoglobulins (Dako, F0479) at a 1:50 dilution for 1 h, both at 4 °C. Beads were washed three times with PBS and analyzed by flow cytometry. Using the standard curve generated by the calibration beads, the MFI of W6/32 binding to HLA-C–conjugated Dynabeads was used to determine the number of HLA-C molecules per bead.

### Redirected Antibody-Mediated Degranulation Assays.

P815 cells were incubated with the following mAbs alone or in combinations at 10 μg/mL for 20 min at room temperature: IgG (MOPC-21; BD Biosciences, 554121), anti-hNKp46 (9-E2; BD Biosciences, 557911), anti-h2B4 (C1.7; Beckman Coulter, IM1607), anti-hCD16 (3G8; BD Pharminogen, 555403), and anti-hKIR2DS4 (179317; R&D Systems, MAB1847). Antibody-coated cells were mixed with resting NK cells in the presence of 1 μL of BV421 anti-CD107a mAb for 2 h at 37 °C (H4A3; Biolegend, 328626). Cells were then stained with mAbs to KIR2DS4 (Miltenyi Biotec; JJC11.6, 130-099-963) and KIR2DL1/S1 (Beckman Coulter; EB6, A22332).

### Retroviral Transduction of 221 Cells with Plasmids Encoding Peptide P2-AV or P2-AW.

The peptides P2-AV and P2-AW were expressed in 221-C*05:01 cells using the PresentER system as described (32). The PresentER plasmid encoding a signal peptide from mouse mammary tumor virus envelope protein, followed by the SIINFEKL epitope, followed by mCherry, was obtained from Addgene (102945), a kind gift of D. Scheinberg, Sloan Kettering Institute, New York, NY (32). The SIINFEKL epitope is encoded by a DNA cassette flanked by mutually exclusive restriction sites for the enzyme SfiI. DNA oligos encoding P2-AV (GGCCGTATTGGCCCCGCCACCTGTGAGCGGGATCATCGACAAGTCCGGCGCCGTGGTGTAAGGCCAAACAGGCC) or P2-AW (GGCCGTATTGGCCCCGCCACCTGTGAGCGGGATCATCGACAAGTCCGGCGCCTGGGTGTAAGGCCAAACAGGCC) with 5′ and 3′ mutually exclusive restriction sites for the enzyme SfiI were synthesized by Integrated Device Technology, Inc. (IDT). Oligos were amplified using PresentER forward (CGACTCACTATAGGGCCGTATTGGCC) and PresentER reverse (AGTGATTTCCGGCCTGTTTGGCC) primers and cloned into plasmid 102945 following SfiI digestion. 293T Phoenix amphoteric cells in 100-mm plates were transfected with PresentER-P2-AV or PresentER-P2-AW. Virus was collected at 48 h and concentrated with Peg-IT (System Bio) for 48 h at 4 °C. A total of 3 × 10^6^ 221-C*05:01 cells were spinoculated with virus in six-well plates at 1,000 × *g* at 32 °C for 2 h with 4 μg/mL polybrene. Two days later, transduced cells were selected with puromycin at 0.5 μg/mL.

### RecA Protein Sequence Analysis.

RecA protein sequences were downloaded from the GenBank. Sequences were searched with the motif xxD/ExxGxWp9 (x = any residue, p9 = F, L, I, M, V). Duplicate species were removed from returned sequences, and the number of unique species and genera was enumerated for each motif.

### KIR and HLA Gene Frequencies.

Allele frequencies of HLA-C*05:01, HLA-C*04:01, and HLA-C*16:01 and the frequency of individuals carrying KIR2DS4-fl (KIR2DS4*001) and the KIR2DS4-del allele (KIR2DS4*003) were obtained from the Allele Frequency Net Database (40) (http://www.allelefrequencies.net/). The KIR allele frequencies from nine populations with high-resolution KIR sequencing were from the Allele Frequency Net Database (http://www.allelefrequencies.net/), from published studies (52, 66–70), and kindly provided by Paul Norman, (University of Colorado, Denver, CO).

### Analysis of Peptides Eluted from HLA-C.

A total of 2,036 9-mer peptides eluted from HLA-C*05:01 collected from three studies were used to analyze the frequency of amino acids at peptide p8 of 9-mers (10, 30, 31). To compare the frequency of Trp usage in 9-mer peptides eluted from 14 HLA-C allotypes, sequences from one study were used (30).

### Flow Cytometry.

Data were acquired on a LSRII or X-20 Fortessa (BD Biosciences). Data were exported as FCS files and analyzed using FlowJo software (TreeStar, version 10). Compensation for multicolor experiments was set using single mAb-stained beads, and cytometer setup and tracking beads were run daily.

### Human Donors.

Peripheral blood samples from healthy US adults were obtained from the NIH Department of Transfusion Medicine under an NIH Institutional Review Board-approved protocol (99-CC-0168) with informed consent.

### Statistical Analysis.

All statistical analysis was carried out in GraphPad PRISM (version 5.0).

## Supplementary Material

Supplementary File

Supplementary File
