# Supplementary material for: Human NK cell receptor KIR2DS4 detects a conserved bacterial epitope presented by HLA-C
Source: Proc Natl Acad Sci U S A. 2019 May 28;116(26):12964–73. doi: 10.1073/pnas.1903781116 (PMC6601252; doi:10.1073/pnas.1903781116)
Supplement: Supplementary File [file pnas.1903781116.sapp.pdf]

THE HUMAN NK CELL RECEPTOR KIR2DS4 DETECTS A CONSERVED BACTERIAL EPITOPE PRESENTED BY HLA-C

Malcolm J. W. Sim[1,2], Sumati Rajagopalan[1], Daniel M. Altmann[3], Rosemary J. Boyton[3], Peter D. Sun[2] and Eric O. Long[1*]

[1]Molecular and Cellular Immunology Section, [2]Structural Immunology Section, Laboratory of Immunogenetics, National Institute of Allergy and Infectious Diseases, National Institutes of Health, Rockville, MD, USA. [3]Lung Immunology Group, Department of Medicine, Imperial College London, UK.

*Corresponding author

SI Appendix: Figures S1-7.

www.pnas.org/cgi/doi/10.1073/pnas.1903781116

Figure S1.

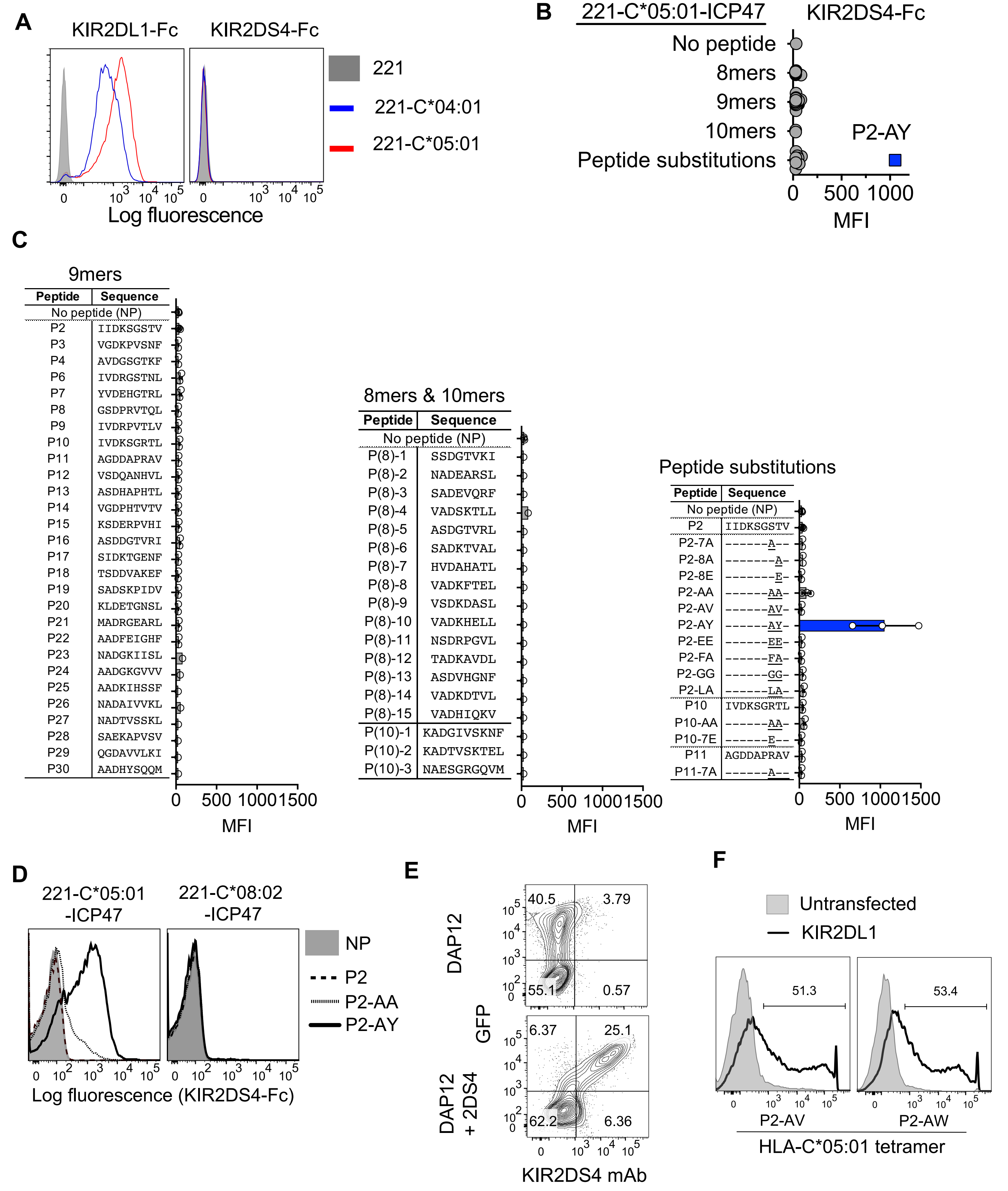
A
KIR2DL1-Fc
KIR2DS4-Fc
221
221-C*04:01
221-C*05:01
Log fluorescence
B
221-C*05:01-ICP47
KIR2DS4-Fc
No peptide
8mers
9mers
10mers
Peptide substitutions
P2-AY
MFI
C
9mers
Peptide Sequence
No peptide (NP)
P2 IIDKSGSTV
P3 VGDKPVSNF
P4 AVDGSGTKF
P6 IVDRGSTNL
P7 YVDEHGTRL
P8 GSDPRVTQL
P9 IVDRPVTLV
P10 IVDKSGRTL
P11 AGDDAPRAV
P12 VSDQANHVL
P13 ASDHAPHTL
P14 VGDPHTVTV
P15 KSDERPVHI
P16 ASDDGTVRI
P17 SIDKTGENF
P18 TSDDVAKEF
P19 SADSKPIDV
P20 KLDETGNSL
P21 MADRGEARL
P22 AADFEIGHF
P23 NADGKIISL
P24 AADGKGVVV
P25 AADKIHSSF
P26 NADAIVVKL
P27 NADTVSSKL
P28 SAEKAPVSV
P29 QGDAVVLKI
P30 AADHYSQQM
MFI
8mers & 10mers
Peptide Sequence
No peptide (NP)
P(8)-1 SSDGTVKI
P(8)-2 NADEARSL
P(8)-3 SADEVQRF
P(8)-4 VADSKTLL
P(8)-5 ASDGTVRL
P(8)-6 SADKTVAL
P(8)-7 HVDAHATL
P(8)-8 VADKFTEL
P(8)-9 VSDKDASL
P(8)-10 VADKHELL
P(8)-11 NSDRPGVL
P(8)-12 TADKAVDL
P(8)-13 ASDVHGNF
P(8)-14 VADKDTVL
P(8)-15 VADHIQKV
P(10)-1 KADGIVSKNF
P(10)-2 KADTVSKTEL
P(10)-3 NAESGRGQVM
MFI
Peptide substitutions
Peptide Sequence
No peptide (NP)
P2 IIDKSGSTV
P2-7A ------A--
P2-8A -------A-
P2-8E -------E-
P2-AA ------AA-
P2-AV ------AV-
P2-AY ------AY-
P2-EE ------EE-
P2-FA ------FA-
P2-GG ------GG-
P2-LA ------LA-
P10 IVDKSGRTL
P10-AA ------AA-
P10-7E ------E--
P11 AGDDAPRAV
P11-7A ------A--
MFI
D
221-C*05:01 -ICP47
221-C*08:02 -ICP47
NP
P2
P2-AA
P2-AY
Log fluorescence (KIR2DS4-Fc)
E
DAP12
GFP
DAP12 + 2DS4
40.5
3.79
55.1
0.57
6.37
25.1
62.2
6.36
KIR2DS4 mAb
F
Untransfected
KIR2DL1
51.3
53.4
P2-AV
P2-AW
HLA-C*05:01 tetramer

Figure S1. Discovery of a peptide:HLA-C*05:01 complex binding to KIR2DS4. **(A)** KIR2DL1-Fc and KIR2DS4-Fc binding to 221, 221–C*04:01 and 221–C*05:01 cells. **(B, C)** KIR2DS4-Fc binding to 221–C*05:01–ICP47 cells loaded with no peptide (NP) or 28 9mer peptides, 15 8mer peptides, 3 10mer peptides and 13 9mer peptides with amino acid substitutions at positions 7 & 8. Data are summarized in (B) and peptide sequences are shown in (C). **(D)** KIR2DS4-Fc binding to 221–C*05:01–ICP47 and 221–C*08:02–ICP47cells loaded with P2, P2-AA and P2-AY or NP**. (E)** Flow cytometry bi-plots displaying KIR2DS4 mAb binding to 293T cells transfected with separate vectors (pIRES2-eGFP) containing cDNA encoding DAP12 and KIR2DS4, or DAP12 only. Binding of anti-KIR2DS4 mAb to 293T cells transfected with DAP12 and KIR2DS4 cDNA or DAP12 cDNA only. **(F)** Binding of HLA-C*05:01 tetramers refolded with P2-AV and P2-AW to untransfected 293T cells or 293T cells transfected with KIR2DL1.

Figure S2.

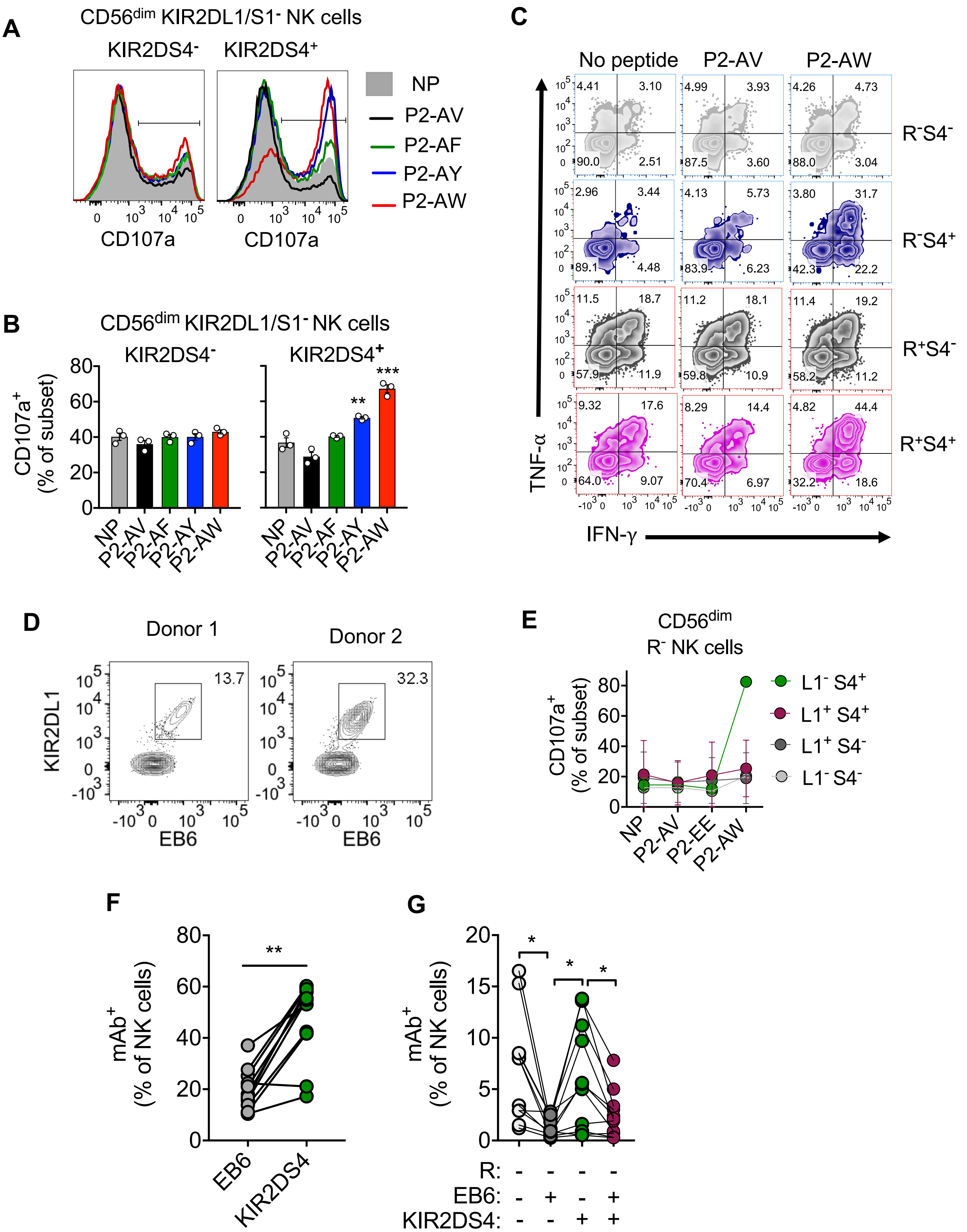
A
CD56dim KIR2DL1/S1- NK cells
KIR2DS4-
KIR2DS4+
CD107a
NP
P2-AV
P2-AF
P2-AY
P2-AW
B
CD56dim KIR2DL1/S1- NK cells
KIR2DS4-
KIR2DS4+
CD107a+ (% of subset)
**
***
C
No peptide
P2-AV
P2-AW
R-S4-
R-S4+
R+S4-
R+S4+
TNF-α
IFN-γ
4.41
3.10
90.0
2.51
4.99
3.93
87.5
3.60
4.26
4.73
88.0
3.04
2.96
3.44
89.1
4.48
4.13
5.73
83.9
6.23
3.80
31.7
42.3
22.2
11.5
18.7
57.9
11.9
11.2
18.1
59.8
10.9
11.4
19.2
58.2
11.2
9.32
17.6
64.0
9.07
8.29
14.4
70.4
6.97
4.82
44.4
32.2
18.6
D
Donor 1
Donor 2
KIR2DL1
EB6
13.7
32.3
E
CD56dim
R- NK cells
CD107a+ (% of subset)
L1- S4+
L1+ S4+
L1+ S4-
L1- S4-
NP
P2-AV
P2-EE
P2-AW
F
mAb+ (% of NK cells)
**
EB6
KIR2DS4
G
mAb+ (% of NK cells)
*
R:
EB6:
KIR2DS4:

Figure S2. Stimulation of KIR2DS4^{+} cells by peptide:HLA-C*05:01 complexes. (A, B) Expression of CD107a on CD56^{dim} KIR2DL1^{-} NK cells in response to 221–C*05:01–ICP47 cells loaded with P2-AV, P2-AF, P2-AY and P2-AW or no peptide (NP). NK cells are gated as CD56^{dim} KIR2DL1/S1^{-}. **(C)** Flow cytometry bi-plots displaying expression of IFN-γ and TNF-α on NK cell subsets defined in (Fig. 2A) in response to 221–C*05:01–ICP47 cells loaded with P2-AV, P2-AW, no peptide or no target cells. Data are from one representative experiment out of 3 independent experiments. **(D)** Flow cytometry bi-plots displaying staining of CD56^{dim} NK cells from two donors with a KIR2DL1 specific mAb (143211) and a KIR2DL1/S1 (EB6) mAb. **(E)** Expression of CD107a on CD56^{dim} R^{-} NK cells in response to 221–C*05:01–ICP47 cells loaded with P2-AV, P2-EE and P2-AW or no peptide (NP). NK cells were gated as into four subsets based on expression of KIR2DS4 (S4) and KIR2DL1 (L1). R^{-} NK cells are defined by lack of KIR2DL2/L3/S2 (clone GL183), KIR3DL1/3DS1 (clone p70) and NKG2A (clone Z199). **(F)** Proportion of CD56^{dim} NK cells from 11 donors which express KIR2DL1/S1 (EB6) or KIR2DS4. **(G)** Proportion of CD56^{dim} R^{-} NK cells from 11 donors which express either KIR2DL1/S1 (EB6) or KIR2DS4 or both.

Figure S3.

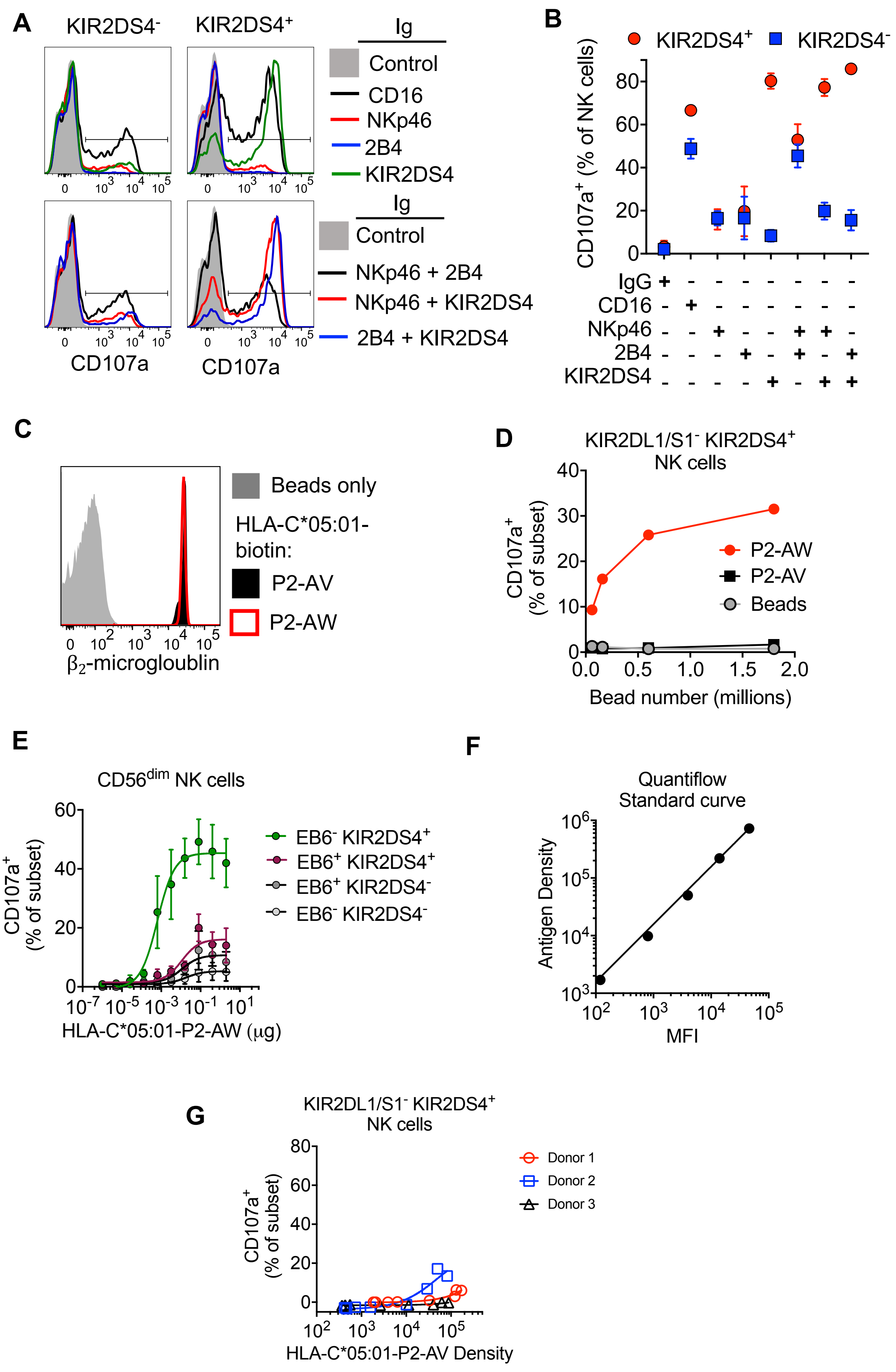
A
KIR2DS4-
KIR2DS4+
Ig
Control
CD16
NKp46
2B4
KIR2DS4
Ig
Control
NKp46 + 2B4
NKp46 + KIR2DS4
2B4 + KIR2DS4
CD107a
CD107a
B
KIR2DS4+
KIR2DS4-
CD107a+ (% of NK cells)
IgG
CD16
NKp46
2B4
KIR2DS4
C
Beads only
HLA-C*05:01-biotin:
P2-AV
P2-AW
β2-microgloublin
D
KIR2DL1/S1- KIR2DS4+ NK cells
CD107a+ (% of subset)
P2-AW
P2-AV
Beads
Bead number (millions)
E
CD56dim NK cells
CD107a+ (% of subset)
EB6- KIR2DS4+
EB6+ KIR2DS4+
EB6+ KIR2DS4-
EB6- KIR2DS4-
HLA-C*05:01-P2-AW (μg)
F
Quantiflow Standard curve
Antigen Density
MFI
G
KIR2DL1/S1- KIR2DS4+ NK cells
CD107a+ (% of subset)
Donor 1
Donor 2
Donor 3
HLA-C*05:01-P2-AV Density

Figure S3. KIR2DS4 is a stronger activator of NK cells than NKp46 or 2B4. (A, B) Expression of CD107a on NK cells in response to P815 cells pre-incubated with specific mAbs to CD16, NKp46, 2B4, KIR2DS4 and combinations of mAbs as indicated. NK cells were gated as KIR2DS4^{+} or KIR2DS4^{-} and data from three independent donors are summarized in B. **(C)** Flow cytometry histograms displaying binding of anti-β_2M mAb to streptavidin Dynabeads only and Dynabeads conjugated to biotinylated HLA-C*05:01 refolded with P2-AV or P2-AW. **(D)** Expression of CD107a on CD56^{dim} KIR2DL1/S1^{-} KIR2DS4^{+} NK cells in response to an increasing number of unconjugated Dynabeads or Dynabeads conjugated to HLA-C*05:01 refolded with P2-AW or P2-AV. **(E)** Expression of CD107a on NK cells in response to Dynabeads conjugated to HLA-C*05:01 refolded with P2-AW. CD56^{dim} NK cells were gated into four subsets based on the expression of KIR2DS4 and KIR2DL1/S1 (EB6). **(F)** Flow cytometry standard curve to determine antigen density from MFI. Calibration beads conjugated to a defined number of mouse immunoglobulins (antigen density) were stained with FITC conjugated polyclonal goat anti-mouse F(ab')$_2$. **(G)** Expression of CD107a on CD56^{dim} KIR2DL1/S1^{-} KIR2DS4^{+} NK cells from 3 donors after stimulation with Dynabeads bearing increasing antigen densities of biotinylated HLA-C*05:01 refolded with P2-AV.

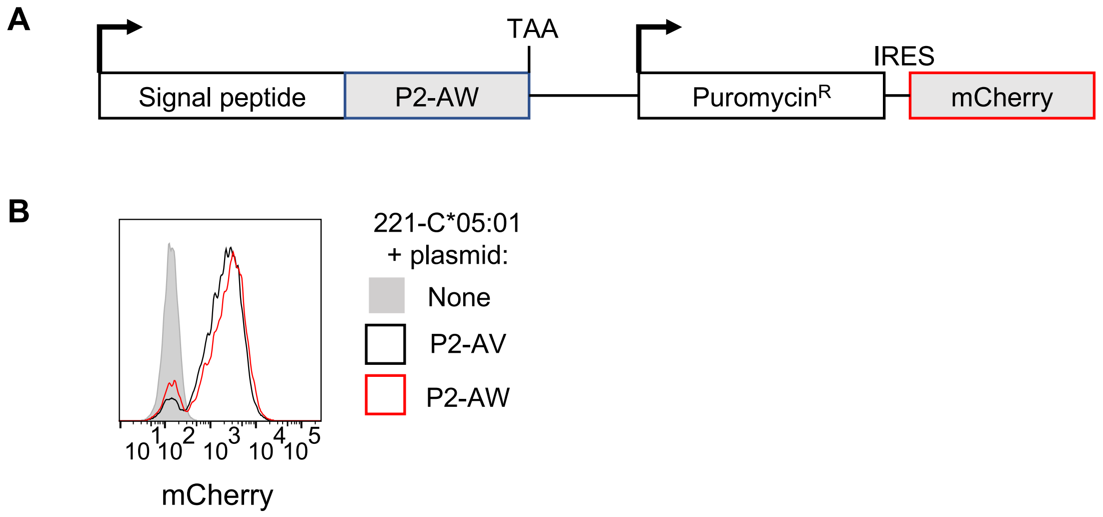

Figure S4. Retroviral transduction of 221-C*05:01 cells with plasmids encoding P2-AV and P2-AW. (A) The plasmid encoding P2-AW includes a signal peptide sequence fused to P2-AW. Arrows indicate translation start site, TAA = stop codon, IRES = internal ribosomal entry sequence. **(B)** Expression of mCherry in in 221–C*05:01 cells after transduction with P2-AV and P2-AW expressing plasmids.

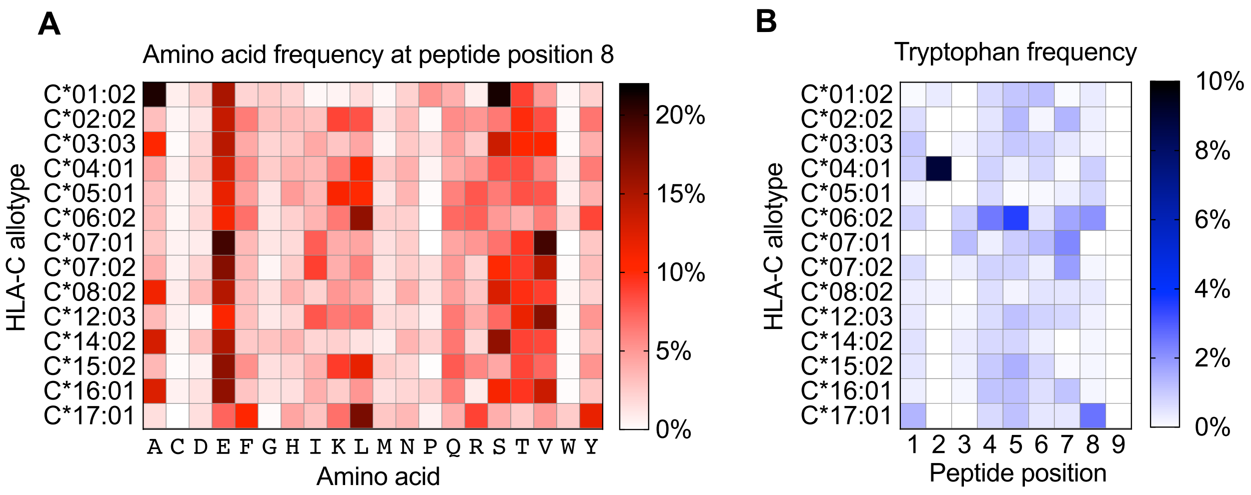

Figure S5. Analysis of peptides eluted from HLA-C. (A) Frequency of individual amino acids at position 8 in all 9mer peptides eluted and sequenced from 14 HLA-C allotypes. The number of peptides eluted ranged from 310 (C*07:01) to 1899 (C*16:01) and were from a previous study (1). **(B)** Frequency of tryptophan at peptide positions 1 to 9 from all 9mer peptides eluted and sequenced from 14 HLA-C allotypes.

Figure S6.

A

Bacterial species	1	2	3	4	5	6	7	8	9	Bacterial species	1	2	3	4	5	6	7	8	9
Pseudomonas fluorescens	F	V	E	K	S	G	A	W	Y	*Methylobacillus flagellatum*	L	I	E	K	A	G	A	W	Y
Streptomyces ambofaciens	F	V	R	K	A	G	A	W	Y	*Proteus vulgaris*	L	I	E	K	A	G	A	W	Y
Streptomyces lividans	F	V	R	K	A	G	A	W	Y	*Yersinia pestis*	L	I	E	K	A	G	A	W	Y
Streptomyces vinaceus	F	V	R	K	A	G	A	W	Y	*Legionella pneumophila*	L	I	E	K	S	G	A	W	Y
Mycobacterium leprae	F	V	R	K	S	G	S	W	F	*Myxococcus xanthus*	L	I	E	K	S	G	S	Y	F
Chlamydia trachomatis	I	I	D	K	K	G	S	W	F	*Borrelia burgdorferi*	L	I	Q	K	T	G	S	W	Y
Treponema pallidum	I	I	E	K	K	G	A	W	Y	*Mycobacterium tuberculosis*	L	I	R	K	S	G	A	W	F
Rickettsia prowazekii	I	I	E	K	S	G	S	W	F	*Azotobacter vinelandii*	L	L	E	K	S	G	A	W	Y
Mycoplasma mycoides	I	I	E	K	S	G	V	W	Y	*Erwinia carotovora*	L	V	E	K	A	G	A	W	Y
Streptococcus pneumoniae	I	I	K	K	A	G	A	W	Y	*Proteus mirabilis*	L	V	E	K	A	G	A	W	Y
Deinococcus radiodurans	I	I	K	K	A	G	S	F	Y	*Vibrio anguillarum*	L	V	E	K	A	G	A	W	Y
Lactococcus lactis	I	I	K	K	S	G	A	W	F	*Xanthomonas oryzae*	L	V	E	K	A	G	A	W	Y
Bacteroides fragilis	I	I	K	K	S	G	S	W	Y	*Haemophilus influenzae*	L	V	E	K	S	G	A	W	Y
Neisseria gonorrhoeae	I	I	N	K	S	G	A	W	Y	*Methylomonas clara*	L	V	E	K	S	G	A	W	Y
Anabaena variabilis	I	L	L	R	K	G	A	W	Y	*Pseudomonas aeruginosa*	L	V	E	K	S	G	A	W	Y
Burkholderia cepacia	I	V	D	K	A	G	A	W	Y	*Pseudomonas putida*	L	V	E	K	S	G	A	W	Y
Campylobacter jejuni	I	V	D	K	S	G	A	W	F	*Acholeplaasma laidlawii*	L	V	N	K	S	G	A	W	Y
Helicobacter pylori	I	V	D	K	S	G	A	W	L	*Serratia marcescens*	M	I	E	K	A	G	A	W	Y
Staphylococcus aureus	I	V	D	K	S	G	A	W	Y	*Vibri cholerae*	M	V	E	K	S	G	A	W	Y
Thermotogoa maritima	I	V	D	R	K	G	S	W	Y	*Acidiphilium facilis*	N	V	E	K	S	G	G	W	Y
Acetobacter polyoxogenes	I	V	E	K	S	G	A	W	F	*Thermus aquaticus*	V	I	E	K	A	G	S	W	F
Agrobacterium tumefaciens	I	V	E	K	S	G	A	W	F	*Thermus thermophilus*	V	I	E	K	A	G	S	W	F
Rhizobium leguminosarum	I	V	E	K	S	G	A	W	F	*Synechococcus strain 7942*	V	I	L	G	K	G	A	W	Y
Rhizobium meliloti	I	V	E	K	S	G	A	W	F	*Aquifex pyrophilus*	V	I	T	K	S	G	S	W	Y
Thiobacillus ferrooxidnas	I	V	E	K	S	G	A	W	Y	*Synechococcus strain 7002*	V	I	T	R	K	G	A	W	Y
Myxococcus xanthus 2	I	V	E	K	S	G	S	W	F	*Mycoplasma pulmonis*	V	L	Q	K	K	G	A	W	F
Corynebacterium glutamicum	I	V	K	K	S	G	S	W	F	*Rhodobacter capsulatus*	V	V	A	K	S	G	A	W	Y
Acinetibacter calcoaceticus	I	V	Q	K	A	G	A	W	Y	*Bordetella pertussis*	V	V	D	K	S	G	A	W	Y
Clostridium perfringens	I	V	Q	K	S	G	A	W	F	*Brucella abortus*	V	V	E	K	S	G	A	W	F
Bacillus subtilis	I	V	Q	K	S	G	S	W	Y	*Rhodobacter sphaeroides*	V	V	E	K	S	G	S	W	Y
Escherichia coli	L	I	E	K	A	G	A	W	Y	*Aquaspirillum magnetotacticum*	V	V	K	K	S	G	A	W	F
Enterbacter agglomerans	L	I	E	K	A	G	A	W	Y	**P2-AW**	I	I	D	K	S	G	A	W	V

B

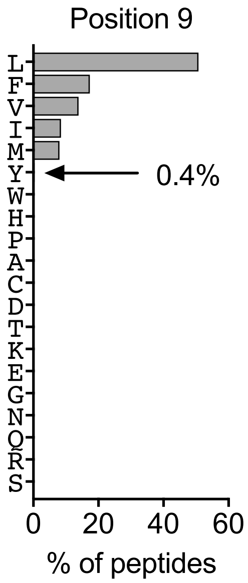

C

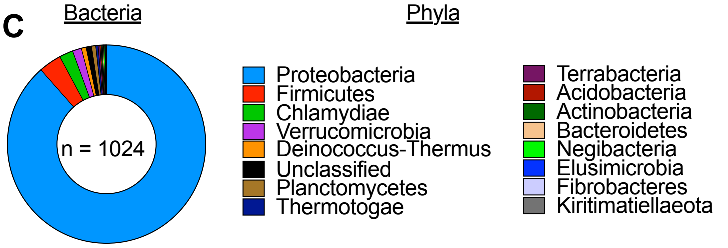

D

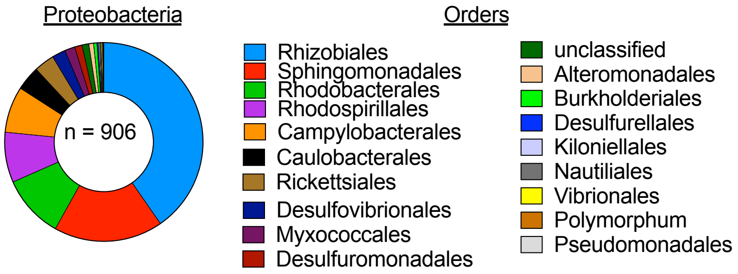

Figure S6. A conserved sequence in RecA contains KIR2DS4 binding peptides.

(A) Individual $RecA_{283\text{-}291}$ sequences from 63 species of bacteria aligned in a previous study (2). The highly conserved region at position 6-8 is colored in blue and position 3, essential for binding to HLA-C*05:01 is colored in red. The P2-AW sequence is included for comparison. **(B)** Frequency of individual amino acids at position 9 in peptides eluted and sequenced from HLA-C*05:01. **(C)** Relative abundance of bacterial species (n=1024) in each of the 16 Phyla, which carry a predicted epitope in RecA for binding to HLA-C*05:01 and KIR2DS4. **(D)** Relative abundance of Proteobacteria species (n=906) in each of the 19 Orders, which carry a predicted epitope in RecA for binding to HLA-C*05:01 and KIR2DS4.

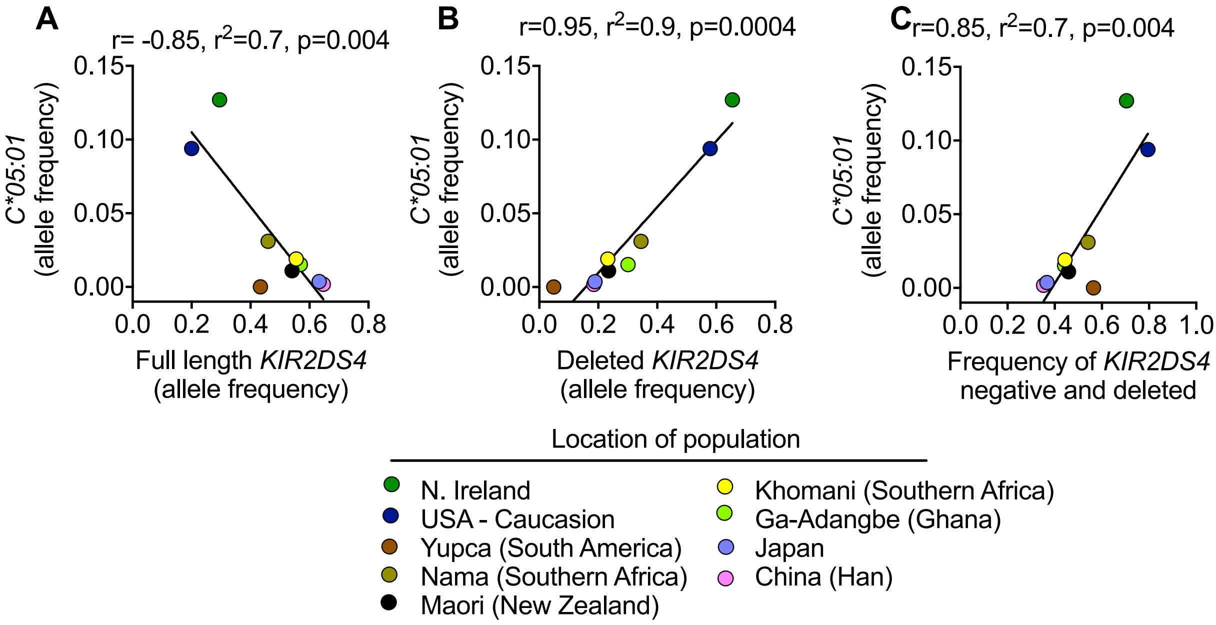

Figure S7. Allele frequency of functional KIR2DS4 is negatively correlated with its ligand HLA-C*05:01. The allele frequency of HLA-C*05:01 in 9 populations is correlated with the frequency of three KIR2DS4 genotypes; KIR2DS4-fl **(A)**, KIR2DS4-del **(B)** and the sum of KIR2DS4-del and those with no KIR2DS4 gene (KIR2DS4-neg; **C**).

SI Appendix References

(1) Di Marco M*, et al.* (2017) Unveiling the Peptide Motifs of HLA-C and HLA-G from Naturally Presented Peptides and Generation of Binding Prediction Matrices. *J Immunol* 199(8):2639-2651.

(2) Karlin S & Brocchieri L (1996) Evolutionary conservation of RecA genes in relation to protein structure and function. *J Bacteriol* 178(7):1881-1894.

Additional files: Supplementary Dataset 1.
